# Safety Profile of Vitamin D in Italy: An Analysis of Spontaneous Reports of Adverse Reactions Related to Drugs and Food Supplements

**DOI:** 10.3390/jcm12144726

**Published:** 2023-07-17

**Authors:** Valentina Maggini, Giada Crescioli, Ilaria Ippoliti, Eugenia Gallo, Francesca Menniti-Ippolito, Adelaide Chiaravalloti, Vittorio Mascherini, Roberto Da Cas, Simona Potenza, Giulia Gritti, Maria Teresa Galiulo, Laura Sottosanti, Alfredo Vannacci, Niccolò Lombardi, Fabio Firenzuoli

**Affiliations:** 1 Research and Innovation Center in Phytotherapy and Integrated Medicine-CERFIT, Referring Center for Phytotherapy of Tuscany Region, Careggi University Hospital, 50141 Florence, Italy; eugenia.gallo@unifi.it (E.G.); adelaide.chiaravalloti@gmail.com (A.C.); mascheriniv@aou-careggi.toscana.it (V.M.); fabio.firenzuoli@unifi.it (F.F.); 2 Department of Neurosciences, Psychology, Drug Research and Child Health, Section of Pharmacology and Toxicology, University of Florence, 50141 Florence, Italy; giada.crescioli@unifi.it (G.C.); niccolo.lombardi@unifi.it (N.L.); 3 National Centre for Drug Research and Evaluation, National Institute of Health, 00161 Rome, Italy; ilaria.ippoliti@iss.it (I.I.); francesca.menniti@iss.it (F.M.-I.); roberto.dacas@iss.it (R.D.C.); 4 General and Clinical Phytotherapy, Department of Experimental and Clinical Medicine, University of Florence, 50141 Florence, Italy; 5 Italian Medicines Agency, 00187 Rome, Italy; s.potenza@aifa.gov.it (S.P.); g.gritti.ext@aifa.gov.it (G.G.); m.galiulo@aifa.gov.it (M.T.G.); l.sottosanti@aifa.gov.it (L.S.)

**Keywords:** vitamin D, adverse reaction, safety, phytovigilance, pharmacovigilance, COVID-19

## Abstract

Vitamin D (VitD) is largely used in Italy, often inappropriately; thus, an evaluation of its safety is a crucial issue. This study analyses the adverse reactions (ARs) associated with the use of products containing VitD (VitDps) reported to the Italian National Pharmacovigilance and Phytovigilance networks. From March 2002 to August 2022, a total of 643 and 127 reports concerning 903 and 215 ARs were retrieved from Pharmacovigilance and Phytovigilance networks, respectively. Overall, 332 (29.6%) ARs were classified as serious, and the most described ones were hypercalcaemia, renal failure and tachycardia. Serious AR risk was significantly higher for subjects using more than four concomitant products (OR 2.44 [95% CI 1.30–4.60]) and VitD doses higher than 1000 IU/day (OR 2.70 [95% CI 1.30–5.64]). In Italy, there was a modest decrease in AR reporting, despite the slightly increased use of VitD during the COVID-19 pandemic. To the best of our knowledge, this is the first study describing all VitDps-related ARs observed in the Italian general population. Since underreporting is the main limitation of the safety reporting systems, the necessity to continue ARs monitoring, also using real-world data on VitDps prescription, use and outcome patterns is highlighted.

## 1. Introduction

Vitamin D3 (cholecalciferol) is produced from 7-dehydrocholesterol by the ultraviolet (UV) light radiation in the skin and is also contained in several foods; alternatively, UVB irradiation produces the analogue vitamin D2 (ergocalciferol, often used for fortification) in plants and fungi. Vitamin D (VitD) regulates calcium and phosphorus homeostasis, promoting musculoskeletal health and osteoid tissue mineralization [1]. VitD deficiency (defined as a serum circulating 25-hydroxyvitamin D, 25(OH)D, below 20 ng/mL (50 nmol/L)) is widespread worldwide and VitD supplementation is recommended for bone health in deficient and at-risk (e.g., older or obese persons, pregnant or postmenopausal women) patients [2,3]. On the other hand, VitD supplementation was not associated with a significantly lower risk of total, nonvertebral or hip fractures than placebo in a trial that enrolled 25,871 generally healthy participants not selected for VitD deficiency or osteoporosis [4], and did not show any improvement in bone mineral density in healthy premenopausal women [5,6]. Considering the evidence from these trials, in February 2023, the Italian Medicines Agency (AIFA) updated the appropriate prescription criteria of Note 96 (a regulatory tool that defines the therapeutic indications for which VitD can be reimbursed within the Italian National Health Service) for VitD supplementation (cholecalciferol, cholecalciferol/calcium and calcifediol) for the “prevention and treatment of VitD deficiency in adult subjects (>18 years of age)” [7]. In the absence of specific at-risk conditions, the reimbursement criteria for VitD use is restricted to subjects with a level of 25(OH)D below 12 ng/mL. On the other hand, the VitD receptor (VDR) is a transcription factor regulating cell-specific genes in most tissues, probably impacting multiple biological targets [8,9]. Therefore, many randomized controlled trials on VitD supplementation for disease prevention or treatment have been conducted and included in meta-analyses, suggesting a possible beneficial role of VitD supplementation in depression [10], blood lipid profile [11,12], blood pressure in the elderly [13] and glycemic control in prediabetes [14] and type 2 diabetes [15]. Conversely, VitD administration showed no protective effect against stroke [16] and no benefit for endometriosis pain [17] and asthma control [18,19]. Furthermore, many studies indicated that VitD deficiency represented a risk factor for the onset of SARS-CoV-2 infection and COVID-19 severe outcomes, and that a beneficial role of VitD in COVID-19 prevention and treatment was hypothesised [20]. However, two clinical trials (the CORONAVIT study on 6200 adults and the CLOC study on 34,601 adults) failed to identify a role of VitD in the prevention of respiratory infections and COVID-19 [21,22].

Products containing VitD (VitDps) are also marketed as food supplements with dosages comparable to that contained in drugs. In this context, it is important to evaluate the safety profile of VitD, both assumed as a drug and food supplement. A 2014 Cochrane review reports VitD adverse reactions (ARs) such as hypercalcaemia, nephrolithiasis, hypercalciuria, renal insufficiency, gastrointestinal disorders, cardiovascular disorders, psychiatric disorders, skin disorders and cancer. Vitamin D3 combined with calcium significantly increases the risk of nephrolithiasis, and alfacalcidol and calcitriol increase the risk of hypercalcemia [23]. Interestingly, a systematic review and meta-analysis on the use of VitD high doses in children aged 0 to 6 years reported that the ARs associated with the integration are rare and there is no increased risk of severe ARs onset [24]. 

Considering the large attention to VitD, in particular, during the COVID-19 pandemic, post-marketing surveillance is fundamental for the collection of new safety data. Therefore, the purpose of this study was to examine the spontaneous reports of ARs associated with VitDps reported to the Italian Phytovigilance and Pharmacovigilance systems. Finally, we focused on the effects of the COVID-19 pandemic on the reporting of ARs related to VitDps.

## 2. Materials and Methods

Spontaneous reports of suspected ARs associated with VitDps recorded from March 2002 to August 2022 were retrieved from the Italian Phytovigilance and Pharmacovigilance systems, coordinated by the National Institute of Health (ISS) and the Italian Medicines Agency (AIFA), respectively [25,26,27,28,29,30]. The Italian Phytovigilance system collects spontaneous reports of suspected ARs related to CAM (Complementary and alternative medicine), while the Italian National Pharmacovigilance Network collects spontaneous reports of suspected adverse drug reactions (ADRs) related to medicinal products. Spontaneous reports can be paper-based or collected online through two different websites (www.vigierbe.it and www.aifa.gov.it/content/segnalazioni-reazioni-avverse) accessed on 31 August 2022. Ethical approval and patient consent are not required due to the nature of the study, which is based on an analysis of the collected spontaneous reports according to the EU General Data Protection Regulation (GDPR 2016/679). The reports on products containing VitD or synonymous compounds (e.g., cholecalciferol, calcifediol, etc.) in the brand name or chemical composition included in the Phytovigilance system were retrieved. ADR reports of medicinal products sent to AIFA were selected by the following suspected active substances: ergocalciferol (A11CC01), dihydrotachysterol (A11CC02), alfacalcidol (A11CC03), calcitriol (A11CC04), cholecalciferol (A11CC05), calcifediol (A11CC06), cholecalciferol combinations (A11CC55), calcium and cholecalciferol (A12AX*). In order not to count AR reports more than once, duplicates were excluded. Only suspect/interacting VitDps were considered; VitDps reported as concomitant agents were also excluded. We collected patient information (age, sex, medical history, etc.), suspected product(s) (type, dosage and treatment duration), concomitant therapies, description of the AR(s) (signs and symptoms or diagnosis, system organ class (SOC), seriousness, outcome, dechallenge, rechallenge, etc.) and the reporter’s qualification. World Health Organization (WHO) criteria were used to evaluate the seriousness and the outcome of each AR [31]. In particular, a serious adverse reaction is “any untoward medical occurrence that at any dose: results in death, is life-threatening, requires inpatient hospitalisation or prolongation of existing hospitalisation, results in persistent or significant disability/incapacity or important medical events, or is a congenital anomaly/birth defect”. A multidisciplinary group, composed of clinical pharmacologists, toxicologists, pharmacists, epidemiologists and experts in pharmaco- and phytovigilance of the Tuscan Pharmacovigilance and Phytovigilance Regional Center (Tuscany, Italy) evaluated each ADR report calculating the causality assessment according to the WHO criteria [32]. ARs were organized in terms of System Organ Class (SOC) and Preferred Term (PT) according to the Medical Dictionary for Regulatory Activities (MedDRA 24.1) [33]. To investigate differences in the rate of ARs between the two reporting systems, Chi-square or Fisher’s exact tests were performed when appropriate.

The SPC (summary of product characteristics) of medicinal products containing VitD reports a potential interaction between VitD and thiazide diuretics, digoxin and warfarin. Thus, for each AR report, the presence of the aforementioned concomitant medication was evaluated. Each SPC is available at: https://farmaci.agenziafarmaco.gov.it/ accessed on 31 August 2022.

A descriptive analysis of the selected reports was performed including reactions related to drug abuse, therapeutic errors, overdose and product use-related problems. Continuous data were expressed as mean and standard deviation (SD) and categorical variables were expressed as numbers and related percentages.

A comparison between serious and non-serious ARs was performed. Moreover, we compared two study periods according to the reporting of suspected ARs of VitDps: period 1 (pre-COVID-19 pandemic), from 1 May 2017 to 31 December 2019; period 2 (during COVID-19 pandemic) from 1 January 2020 to 31 August 2022. To investigate differences in the rate of AR reports between the two periods, Chi-square or Fisher’s exact tests were performed when appropriate.

A multivariate logistic regression model was used to estimate the odds ratios (ORs) with 95% confidence intervals (CIs) of serious ARs according to age, sex, presence of concomitant medications or other products and duration of treatment. We used STATA v17 software for data analysis and considered the results statistically significant at *p* < 0.05. 

## 3. Results

From March 2002 to 31 August 2022, a total of 127 and 643 AR reports concerning 215 and 903 ADRs to VitDps were collected in the Italian Phytovigilance and Pharmacovigilance databases, respectively.

### 3.1. Demographic and Clinical Data

The median age of subjects was 62 years (range: 4 days–95 years), and 78.2% were females (Table 1). As for motivation for use, osteoporosis (34.1%) was reported only for drugs whereas “integration” was indicated for both food supplements (22.0%) and drugs (29.7%). The VitDps duration of use varied from 1 day to 19 years, with a marked difference for therapies longer than 30 days between food supplements (9.4%) and drugs (28.6%). Forty per cent of patients (n = 311) were also taking other medications. The overall median time from VitDps initiation to AR onset was 6 days, and 60.8% and 69.9% of serious ARs occurred within the first 30 days, respectively, for food supplements and drugs. The most frequently reported suspected products were Dibase^®^ (Abiogen Pharma, Ospedaletto, Pisa, Italy; cholecalciferol, 10,000–300,000 IU/mL; 39.5% of the AIFA number of reports) among registered medications, and Multicentrum^®^ (Haleon Italia, Milan, Italy; vitamins: A, B1, B2, B6, B12, C, D (5 µg/capsule), E, K, biotin, niacin, folic acid, pantothenic acid; minerals: calcium, phosphorus, magnesium, iron, zinc, iodine, chromium, copper, manganese, molybdenum, selenium; 8.7% of the ISS number of reports), including Multicentrum Donna^®^ (10 µg/capsule), Uomo^®^ (10 µg/capsule), Junior^®^ (3 µg/capsule) and Materna^®^ (12.5 µg/capsule), among dietary supplements.

### 3.2. AR Reports from the Italian Phytovigilance System (ISS)

Physicians and pharmacists reported the majority of cases (65.4%), followed by patients (26.8%) (Table 1). Complete resolution or recovery occurred in 53.5% of subjects. Dechallenge was positive in 33.8% of cases, while rechallenge led to the re-occurrence of reactions in 15 cases (11.8%). VitDps dosage was not reported in the Italian Phytovigilance system, and drug abuse was indicated in only one case.

The most frequently reported ARs were vomiting (11.6%), nausea (8.8%) and diarrhoea (7.4%). Moreover, 15 (6.9%) cases of urticaria and/or pruritus were recorded, as well as 9 (4.2%) cases of erythema. Details of each AR report retrieved are described in Table S1.

Overall, 216 ARs were registered (each report could have more than one PT from the same SOC), meaning that each report had a mean of 1.70 reactions. Table 2 shows the 21 SOCs identified, with the class “Gastrointestinal disorders” as the most frequently reported one, identified in 43.5% (n = 94) of the reports, followed by “Skin and subcutaneous tissue disorders”, in 16.7% (n = 36), “General Disorders and administration site conditions” and “Investigations” both in 6.9% (n = 15) of cases.

### 3.3. ADR Reports from the Italian Pharmacovigilance system (AIFA)

Suspected ADRs were mainly reported by physicians and pharmacists (68.4%), followed by patients (22.0%) (Table 1). In terms of outcomes, 65.3% of subjects experiencing ADRs completely recovered/improved. Information on dechallenge was reported in 376 (58.5%) cases and was “positive” in 349 (54.3%). Information on rechallenge was reported in 42 (6.5%) cases and was “positive” in 38 (5.9%).

VitD dosage was available for 417 subjects (it was not reported or not assessable in 172 and 54 cases, respectively). VitDps median daily dose was 880 (10–300,000) IU in 289 reports and it was 7500 (20–400,000) IU/week and 100,000 (10,000–1,000,000) IU/month in 63 and 27 cases, respectively. Cyclic VitD administration was recorded in 11 reports (median 25,000, 10,000–100,000 IU/cycle). Seventeen patients took a single dose (median 25,000, 500–300,000 IU/once) and 3 patients were administered an annual dose of 300,000 IU. Finally, seven patients reported mixed administration (e.g., daily dose plus weekly or monthly dose). Several ADRs were characterized as overdose (0.5%), abuse (1.6%), therapeutic error (2.9%) and off-label or accidental use (0.5%).

The ADRs most reported were urticaria and/or pruritus (8.4%), gastrointestinal pain (6.2%), followed by hypercalcemia (4.1%) and diarrhoea (3.7%). Details of each ADR report retrieved from the Italian Pharmacovigilance network are described in Table S2.

Most ADRs were allocated to the “Gastrointestinal disorders” system organ class (n = 310, 26.7%), followed by “Skin and subcutaneous tissue disorders” (n = 255, 22.0%), “Nervous system disorders” (n = 130, 11.2%) and “Investigations” (n = 94, 8.1%) classes (Table 2).

### 3.4. Seriousness and Causality Assessment

Overall, 172 reports described 332 (29.6%) ARs classified as serious (in 52 cases seriousness was not reported) (Table 2). ARs caused or prolonged the hospitalization and other medically important conditions in 103 (59.9%) and 42 (24.4%) subjects, respectively. Life threatening ARs were reported in 14 (8.1%) cases, disability in 10 (5.8%), birth defects in 1 (0.6%) case and 2 subjects died. Fifty-six (out of 172) cases (32.6%) had complete resolution of the AE and the outcome was unknown in 21.5% of reports.

**Figure 1:**
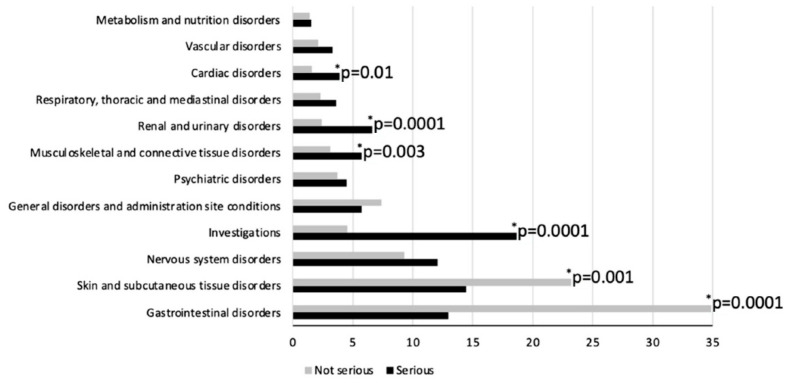
Comparison between overall non-serious and serious adverse reactions (ARs) and adverse drug reactions (ADRs) to products containing vitamin D by SOC terms. Only non-serious and serious reactions with a prevalence >1% are shown.

By analyzing differences at the SOC level, “Gastrointestinal disorders” (34.9% vs. 12.9%; *p* < 0.01) and “Skin and subcutaneous tissue disorders” (23.2% vs. 14.5%; *p* < 0.01) were significantly higher in non-serious reactions (Figure 1). On the other hand, “Investigations disorders” (18.7% vs. 4.5%; *p* < 0.01), “Renal and urinary disorders” (6.6% vs. 2.4%; *p* < 0.01), “Musculoskeletal and connective tissue disorders” (5.7% vs. 3.1%; *p* = 0.03) and “Cardiac disorders” (3.9% vs. 1.6%; *p* < 0.01) were significantly more represented in serious cases.

Observing the individual PTs, the most-described serious ARs of the aforementioned SOCs were hypercalcemia, renal failure and tachycardia (Table 3). Of note, eight subjects experienced both hypercalcemia and renal failure, and one patient reported the simultaneous onset of hypercalcemia and renal and heart failure.

### 3.5. Risk Factors for Serious ARs

The multivariate logistic regression model showed that age and sex did not contribute to the risk of serious ARs onset; conversely, the risk to develop a serious AR was significantly higher for subjects reporting more than four concomitant products (OR 2.44 [95% CI 1.30–4.60]), and for treatment longer than 7 days (OR 2.08 [95% CI 1.12–3.86] for 8–30 days; OR 1.91 [95% CI 1.16–3.15] for more than 30 days). The available data for VitDps dosage and seriousness showed a significantly higher probability of experiencing a serious AR for subjects exposed to doses of VitDps higher than 1000 IU/day (28 vs. 12%). Specifically, a crude OR of 2.70 (1.30–5.64 95% CI) was estimated (Table 4).

### 3.6. VitDps and Pharmacological Interactions

Observing concomitant drugs, thiazide diuretics, digoxin and warfarin resulted to be present in 14, 7 and 19 reports, respectively (Table S3). Twelve patients taking VitD and warfarin showed variations in the international normalization ratio (INR) or prothrombin time. No AR was related to the onset of digoxin toxicity (i.e., arrhythmia), and serious ARs and hypercalcemia were not reported for patients using VitD and thiazide diuretics.

### 3.7. ARs Comparison between Pre-COVID-19 and during COVID-19 Pandemic

**Figure 2:**
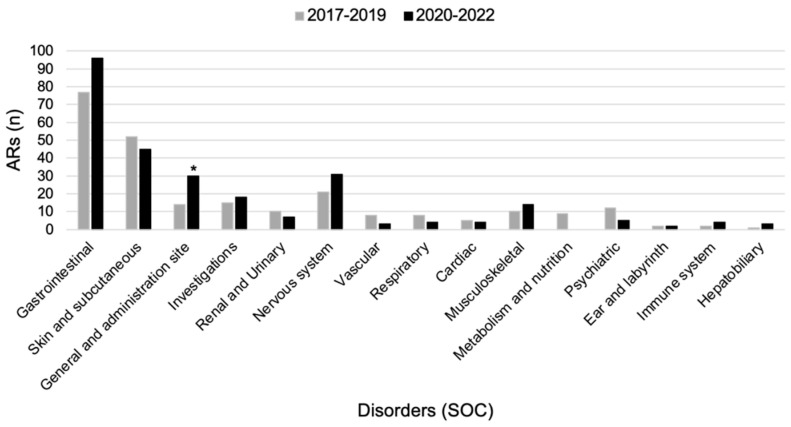
Comparison of ARs and ADRs distribution by System Organ Class (SOC) between pre-COVID–19 and during the COVID–19 pandemic. * *p* < 0.05.

Overall, 288 reports were recorded pre-COVID-19 pandemic (period 1) and during the COVID-19 pandemic (period 2): 155 (53.8%) in period 1 vs. 133 (46.2%) in period 2. The percentage of serious reports slightly decreased in period 2 (27.1% vs. 24.8%; *p* = 0.51). The causality assessment was shown to be “probable” (48.7%) and “possible” (48.7%) for the majority of reports collected in period 1 as well as for those collected in period 2 (50.0% “probable” and 49.1% “possible”, respectively). “Gastrointestinal disorders” (31.3% vs. 36.2%; *p* = 0.26) and “Skin and subcutaneous tissue disorders” (21.1% vs. 17.0%; *p* = 0.22) were the first two SOCs more frequently reported in both periods. ARs belonging to “General disorders” were more frequently reported in period 2 (11.3% vs. 5.7%; *p* = 0.02), otherwise “Metabolism and nutrition disorders” ARs (3.7%) were only reported in period 1 (Figure 2; Table S4). Serious and non-serious ARs distributions by SOC were reported for each year of the two reference periods (Tables S5 and S6).

## 4. Discussion

The present study analyses ARs associated with VitDps reported to the Italian Phytovigilance and Pharmacovigilance systems, with a focus on the effects of the COVID-19 pandemic on spontaneous ARs reporting. From 2002 to August 2022, a total of 770 AR reports following VitDps administration were collected. To the best of our knowledge, this is the first study describing all VitDps-related ARs observed in the Italian general population (all age groups) and comparing the rate before and during the COVID-19 pandemic period.

A systematic review and meta-analysis of randomized controlled trials on the safety of long-term vitamin D2 or D3 supplementation found that VitD does not increase the risk of non-calcemic ARs [34]. Generally, gastrointestinal and dermatological ARs are mild, and reversible [19]. Our results confirmed this evidence, with most ARs (73.8%) non-serious, belonging to gastrointestinal and skin disorders, and completely resolved or recovered in more than 45% of cases.

Real-world data from US poison centres showed a low incidence of serious cases following vitamin D acute exposure which is consistent with data on serious reactions associated with VitD chronic ingestion [35]. The most commonly reported serious ARs were related to cardiac disorders and hypercalcemia [23,24]. Two meta-analyses found a statistically significant association between VitDps supplementation and the occurrence of hypercalcemia and hypercalciuria [36,37]. These data were in line with our study results showing that the SOCs most frequently reported for serious ARs were investigations (i.e., hypercalcemia) and renal and urinary (e.g., renal failure), musculoskeletal (e.g., musculoskeletal pain) and cardiac disorders (e.g., tachycardia). Of note, hypercalcemia is itself responsible for many symptoms ranging from mild gastrointestinal disorders to severe events such as seizures, coma and death [38]. Other hypercalcemia-related symptoms include bone, muscle and joint pain, irregular heartbeat, polyuria and acute renal injury and failure [39]. We observed serious hypercalcemia after supplementation of vitamin D3 or D2 or the active form 1,25(OH)2 D (1,25-dihydroxy vitamin D, calcitriol) or both. Similarly, the onset of vitamin D hypercalcemia is mainly related to the use of calcitriol [40,41]. In fact, calcitriol is immediately absorbed, producing an expedited plasma peak with respect to vitamin D3 regulated by liver activation [42]. Of notice, prolonged hypercalcemia may determine impaired renal function and an increase in renal diseases [40,43]. In our data, we observed 22 serious ARs belonging to the SOC “Renal and urinary disorders”, in particular, renal failure and nephrocalcinosis. Of note, eight subjects experienced both hypercalcemia and renal failure.

In our sample, we also observed serious ARs belonging to the “Cardiovascular disorders” in nine patients (four patients used cholecalciferol and four calcitriol), in particular (reported as PT), tachycardia and chest pain. Notwithstanding, epidemiological studies have reported an association between low vitamin D levels and elevated cardiovascular risk, and available evidence for the role of VitD supplementation in the decrease of the cardiovascular risk is controversial [44]. On the other hand, VitD harmful effects are reported at 25(OH)D levels > 125 nmol/L [45]. Since calcium plays an important role in regulating the duration of cardiac cells action potential [46], the QT interval is affected by the changes in serum calcium concentrations. Specifically, the QT interval is shortened in patients with hypercalcemia [47] and this inverse association has been confirmed in two large samples of the U.S. general population [46]. In this context, it is well known that QT interval shortening is associated with increased risk of arrhythmias, mortality risk and sudden cardiac death [48,49]. Moreover, 1,25(OH)2D is suggested as a negative regulator of the renin–angiotensin system, thus having a potential role in hypertension [50]. Also, VitD-induced hypercalcemia has been associated with vascular calcification [51,52]. Moreover, patients with advanced heart failure taking daily 4000 IU of cholecalciferol-D3 for 3 years showed an increased need for mechanical circulatory support implants with respect to the placebo group [53]. In our sample, a suspected myocardial infarction was reported for one patient taking concomitant cardiovascular medications, presumably related to a previous cardiovascular event. Since the evidence for beneficial vitamin D effects on cardiovascular disease risk is lacking, clinicians should use caution, particularly to manage patients with heart disorders.

### 4.1. Pharmacological Interactions and Dosage

Regarding the use of VitDps in combination with other pharmacological therapies (both medications and/or CAM products) in our sample, 40% of patients were administered with at least another concomitant product. Moreover, subjects who had more than four concomitant products had an increased risk to develop serious ARs. This evidence is well-known for polypharmacy cases, especially in the presence of supplemental products and in frail subjects (i.e., elderly) [25,27,28,54]. The potential for VitDps to interact with certain medications has been investigated. For example, concomitant use of thiazide diuretics and VitD may cause hypercalcemia in the elderly or in subjects with renal impairment or hyperparathyroidism [55], and VitD supplementation may enhance the anticoagulant effect of warfarin reducing warfarin maintenance dose requirement [56], or increase digoxin toxicity [57]. Indeed, we did not observe any serious ARs or hypercalcemia in patients using VitD and thiazide diuretics, but we cannot exclude that several ARs (e.g., gastrointestinal) were related to a higher-than-normal level of serum calcium. Also, in our data, no digoxin toxicity (i.e., arrhythmia) was related to concomitant use of VitD and digoxin. Conversely, an increase in INR or prothrombin time was reported, and in two cases, the physician specifically reported pharmacological interaction. These results were in line with the reported VitD antithrombotic effect as well as with the negative relationship between serum vitamin D status and the risk of venous thromboembolism found in a meta-analysis of observational studies [58]. Moreover, cancer patients treated with high-dose calcitriol showed a lower number of thrombotic events with respect to placebo [59]. Furthermore, the VitD deficiency treatment (oral dose of 50,000 IU vitamin D3) in patients with deep vein thrombosis or pulmonary embolism resulted in the need for lower doses of warfarin to control the INR compared with the control group not receiving VitD [56]. Vitamin D could potentially interfere with warfarin metabolism. In fact, the vitamin D receptor (VDR) was associated with the induction of transcriptional changes of CYP2C9 involved in the reduction of warfarin clearance [60].

Our data showed an association between the risk of developing serious ADRs and VitD dose > 1000 IU/day. However, as recommended by the European Food Safety Authority (EFSA) and Scientific advisory committee on nutrition (SACN), a safety upper limit of 4000 IU/day was consistently accepted for adults and children aged 11–17 years [61]. Since the ARs risk was also reported depending on the age, sex, VitD status of the individuals and the regimen administration, this limit should be investigated and related to the VitD therapeutic window [62]. In fact, data from observational studies evidenced a “V” trend of the curve relating the levels of 25(OH)D and the incidence of negative outcomes (including mortality): the incidence of ARs decreased to a minimum risk when the 25(OH)D level ranged between 20 and 45 ng/mL, then the reaction risk increased [63,64]. In this context, a meta-analysis of 94 cohort studies indicated that daily supplementation with 800 IU/day was adequate to achieve an optimal vitamin D status in adults [65]. Moreover, elevated levels of 25(OH)D were associated with an increased risk of prostate cancer [66] and increasing dietary VitD intake was related to the risk of pancreatic cancer [67]. The D-Health randomized placebo-controlled trial of the effect of oral vitamin D3 supplementation (60,000 IU per month) on mortality reported that VitD administration did not reduce all-cause mortality [68]. Exploratory analyses excluding the first two years of follow-up were consistent with an increased risk of death from cancer (placebo and VitD group mean serum 25(OH)D concentrations were 77 (±25) and 115 (±30) nmol/L, respectively) [68]. In our sample, one physician reported the onset of lung adenocarcinoma in a patient with multiple sclerosis treated with cholecalciferol and fingolimod. Moreover, rare clinical case reports of VitD-associated fatality were described [69,70,71,72], and in our sample, two ADRs with fatal outcomes were retrieved. In our sample, physicians reported the onset of advanced cancer and agranulocytosis (with fatal outcomes) in two patients treated with cholecalciferol. Suspect lung adenocarcinoma was also reported in one patient. In relation to cancer risk, the conclusion of the Italian Medicines Agency [7] can be summarised in the invitation not to exceed physiological 25(OH)D ranges and to prefer low doses considered safe (800–1000 IU per day) [62].

Finally, VitD toxicity is also related to inappropriate prescribing, high-dose dietary supplements use and manufacturing errors [73]. Moreover, the lack of accuracy in manufacturing and labelling is a globally recognized problem [74]. We reported three cases of vitamin D intoxication with severe hypercalcemia, [72] where the patients received a formulation with a VitD concentration 880 times higher than on the product’s label [75].

### 4.2. COVID-19 Pandemic Influence

In Italy, there was a modest decrease in AR reporting, despite the slightly potential increased use of VitD during the COVID-19 pandemic [76]. Probably, healthcare professionals (strongly captured by the COVID-19 emergency) focused on the spontaneous ARs reporting of vaccines or specific drugs used for COVID-19 [77] rather than on VitD and all other drugs. During the pandemic, citizen reporting also considerably increased [77]. In any case, our data confirmed general knowledge on VitDps toxicity, and the constant incidence of serious ARs onset during the pandemic context showed that phyto- and pharmacovigilance monitoring is even more worthy after media hype about the role of VitD in COVID-19 prevention and treatment. It should be important to explore self-medication potential hazards along with policymaker countermeasures [78].

### 4.3. Limitations and Strengths

An important issue affecting the spontaneous reporting systems is underreporting, which increased during the pandemic for drugs and food supplements [77]. Moreover, the Phytovigilance system collects spontaneous reports of ARs related to the intake of dietary supplements, herbal preparations and galenic formulations [79], but we have found only one report for VitD galenic formulations which are especially prescribed to administer high doses of VitD to enhance VitD bioavailability and palatability [80]. Also, the analysis of the suspected ARs associated with dietary supplements containing VitD and other ingredients did not permit the establishment of if the reported effects were directly related to VitD, to another component or to the association. Furthermore, demographic and clinical data (i.e., concomitant comorbidities, dosage, etc.) may be lacking in the spontaneous reports. For these reasons, post-marketing surveillance needs to be strengthened and it would be advisable to have homogeneous national surveillance databases to achieve an optimal level of safety evaluation of CAM and medicinal products. Finally, the consumption of medicines in Italy can be assessed/evaluated through the AIFA OsMed annual reports (https://www.aifa.gov.it/en/rapporti-osmed, accessed on 31 August 2022). On the contrary, information on food supplement use in the real-world is difficult to find and, for this reason, ad hoc studies are needed. In fact, information on the prevalence of use (denominator) of these products in the general population could help to better characterise the safety profile of VitD.

In any case, the spontaneous reporting system represents a fundamental tool to identify rare and serious ARs not detected during premarketing clinical phases [81]. Moreover, it can detect safety signals for CAM products, whose efficacy and safety profiles are not normally evaluated with clinical trials [82,83].

## 5. Conclusions

VitDps can be considered generally safe and well tolerated if the therapeutic dose is carefully determined and monitored, paying particular attention (e.g., speaking with healthcare professionals before taking VitDps) if you are in a long-term treatment or are taking other medicines. For example, 25(OH)D and calcium levels should be assessed in at-risk patients (e.g., use of antithrombotics or medications associated with hypercalcemia such as calcium, VitD and thiazide diuretics; concomitant cardiovascular, renal and intestinal diseases) [39,62]. Moreover, future studies should evaluate the safety of VitDps alone or combined with calcium, since calcium supplements without vitamin D are associated with an increased risk of hip fracture [84].

The accumulating evidence on a beneficial role of vitamin D, especially in particular populations, strengthens the need to increase the knowledge of the VitD safety profile to ensure a positive benefit/risk profile evaluation for indications such as prevention and treatment of vitamin D deficiency (e.g., for bone and muscular health) [85]. Thus, the present study points out the necessity to monitor the ARs related to VitDps and to conduct further research, for example, to evaluate the efficacy and safety of preventive and/or therapeutic strategies in population subgroups, such as individuals with a combination of low vitamin D status with specific gene variants and/or certain nutrition and lifestyle factors [86,87]. Also, real-world data from electronic healthcare databases should be used to monitor VitD prescription, use and outcome patterns [88]. In particular, studies evaluating the prevalence of the use of medicines and food supplements containing VitD in the general population are needed to better clarify its safety profile in clinical practice.

## Figures and Tables

**Table 1:** Characteristics of patients reporting ARs and ADRs to CAM and medicinal products containing VitD.

	Italian Phytovigilance System (ISS)			Italian Pharmacovigilance System (AIFA)		
Characteristics	Overall	Serious ^a^	Non-Serious ^a^	Overall	Serious ^b^	Non-Serious ^b^
	n = 127 (%) ^c^	n = 26 (%) ^c^	n = 100 (%) ^c^	n = 643 (%) ^c^	n = 146 (%) ^c^	n = 468 (%) ^c^
**Age (years; mean ± SD)**	37.8 ± 26.4	43.0 ± 22.8	36.0 ± 27.6	59.9 ± 21.1	59.5 ± 23.1	59.7 ± 20.9
**Sex**						
Male	36 (28.4)	9 (34.6)	27 (27.0)	123 (19.1)	39 (26.7)	82 (17.6)
Female	86 (67.7)	17 (65.4)	68 (68.0)	516 (80.3)	106 (72.6)	383 (81.8)
Not reported	5 (3.9)	-	5 (5.0)	4 (0.6)	1 (0.7)	3 (0.6)
**Comedications**						
≥5 drugs	10 (7.9)	4 (15.4)	6 (6.0)	66 (10.3)	27 (18.5)	37 (7.9)
1–4 drugs	32 (25.2)	11 (42.3)	20 (20.0)	203 (31.6)	31 (21.3)	157 (33.5)
No drugs	6 (4.7)	1 (3.8)	5 (5.0)	322 (50.1)	70 (47.9)	240 (51.3)
Not reported	79 (62.2)	10 (38.5)	69 (69.0)	52 (8.1)	18 (12.3)	34 (7.3)
**Reason of use**						
Vitamin integration ^d^	28 (22.0)	9 (34.7)	19 (19.0)	191 (29.7)	47 (32.2)	143 (30.5)
Adaptogen/Tonic ^e^	7 (5.5)	1 (3.8)	6 (6.0)	-	-	-
Pain	5 (3.9)	1 (3.8)	4 (4.0)	11 (1.7)	2 (1.4)	8 (1.7)
Pregnancy	5 (4.1)	1 (3.8)	3 (3.0)	-	-	-
Menopause	3 (2.4)	1 (3.8)	2 (2.0)	1 (0.2)	-	1 (0.2)
Osteoporosis/Osteopenia	-	-	-	219 (34.1)	32 (21.9)	168 (35.9)
Thyroid-related	-	-	-	19 (2.9)	13 (8.9)	5 (1.1)
Others	19 (14.9)	4 (15.4)	15 (15.0)	26 (4.1)	8 (5.5)	17 (3.6)
Not reported	60 (47.2)	9 (34.7)	51 (51.0)	176 (27.4)	44 (30.1)	126 (27.0)
**Duration of treatment**						
≤7 days	31 (24.4)	5 (19.2)	26 (26.0)	189 (29.4)	33 (22.6)	149 (31.8)
8–30 days	17 (13.4)	6 (23.1)	11 (11.0)	72 (11.2)	11 (7.5)	51 (10.9)
>30 days	12 (9.4)	4 (15.4)	8 (8.0)	184 (28.6)	54 (38.0)	129 (27.6)
Not reported	67 (52.8)	11 (42.3)	55 (55.0)	198 (30.8)	48 (32.9)	139 (29.7)
**Time to onset ^f^**						
≤7 days	48 (39.3)	5 (20)	43 (44.8)	236 (36.5)	36 (24.7)	194 (41.4)
8–30 days	14 (11.4)	5 (20)	9(9.4)	64 (11.2)	13 (8.9)	43 (9.2)
>30 days	14 (11.4)	5 (20)	9 (9.4)	163 (25.3)	53 (36.3)	106 (22.7)
Not reported	46 (37.7)	10 (40)	35 (36.4)	180 (28.0)	44 (30.1)	125 (26.7)
**Reporter qualification**						
Physician	49 (38.6)	20 (76.9)	29 (29.0)	333 (51.8)	85 (58.2)	225 (48.1)
Pharmacist	34 (26.8)	2 (7.7)	32 (32.0)	107 (16.6)	21 (14.4)	86 (18.4)
Patient	34 (26.8)	3 (11.6)	30 (30.0)	141 (22.0)	11 (7.5)	128 (27.3)
Other	9 (7.0)	1 (3.8)	8 (8.0)	62 (9.6)	29 (19.9)	29 (6.2)
Not reported	1 (0.8)	-	1 (1.0)	-	-	-
**Seriousness**						
Hospitalisation/prolongation of existing hospitalisation	-	19 (73.1%)	-	-	84 (57.5%)	-
Other important medical events	-	2 (7.7%)	-	-	40 (27.4%)	-
Life-threatening	-	3 (11.5%)	-	-	11 (7.5%)	-
Persistent or significant disability or incapacity	-	2 (7.7%)	-	-	8 (5.5%)	-
Fatal	-	-	-	-	2 (1.4%)	-
Birth defect	-	-	-	-	1 (0.7%)	-
**Outcomes**						
Recovered/resolved	56 (44.1)	8 (30.8)	48 (48.0)	260 (40.4)	48 (32.9)	196 (41.9)
Recovering/resolving	12 (9.4)	8 (30.8)	4 (4.0)	160 (24.9)	47 (32.2)	112 (23.9)
Recovered with sequelae	2 (1.6)	-	2 (2.0)	10 (1.6)	3 (2.1)	6 (1.3)
Not recovered	9 (7.1)	3 (11.5)	6 (6.0)	66 (10.3)	16 (11.0)	50 (10.7)
Fatal	-	-	-	2 (0.3)	2 (1.3)	-
Unknown	48 (37.8)	7 (26.9)	40 (40.0)	145 (22.5)	30 (20.5)	104 (22.2)
**Dechallenge**						
Positive	43 (33.8)	9 (34.6)	34 (34.0)	349 (54.3)	74 (50.7)	262 (56.0)
Negative	2 (1.6)	-	2 (2.0)	27 (4.2)	8 (5.5)	19 (4.0)
Not reported	82 (64.6)	17 (65.4)	64 (64.0)	267 (41.5)	64 (43.8)	187 (40.0)
**Rechallenge**						
Positive	15 (11.8)	-	15 (15.0)	38 (5.9)	5 (3.3)	31 (6.6)
Negative	1 (0.8)	-	1 (1.0)	4 (0.6)	1 (0.7)	3 (0.6)
Not reported	111 (87.4)	26 (100)	84 (84.0)	601 (93.5)	140 (95.9)	434 (92.8)
**Causality assessment**						
Definite	8 (6.3)	2 (7.7)	6 (6.0)	-	-	-
Probable/likely	58 (45.7)	8 (30.8)	50 (50.0)	240 (37.3)	43 (29.5)	191 (40.8)
Possible	35 (27.5)	13 (50.0)	22 (22.0)	388 (60.3)	97 (66.4)	269 (57.5)
Unlikely	1 (0.8)	-	-	8 (1.3)	4 (2.7)	3 (0.6)
Unassessable/unclassifiable	25 (19.7)	3 (11.5)	22 (22.0)	7 (1.1)	2 (1.4)	5 (1.1)

AR: adverse reaction; ADR: adverse drug reaction; AIFA: Italian Medicines Agency; CAM: complementary and alternative medicine; ISS: National Institute of Health; SD: standard deviation; VitD: vitamin D. ^a^ In one case, seriousness was Not reported. ^b^ In 29 cases, seriousness was Not reported. ^c^ % refers to the total of each column. ^d^ Medicinal products: the cut-off level of 25OHD dosing for initiating therapy is 10–12 ng/mL (or 25–30 nmol/L). ^e^ Dietary supplements: integration and adaptogenic/tonic use are definitions indicating that they are intended to improve or maintain overall health adding to or supplementing the diet. ^f^ Days between starting treatment and AEs onset.

**Table 2:** ARs and ADRs grouped by System Organ Class (SOC) and ordered by total SOC frequency.

System Organ Class (SOC)	ISS ARs n (%) ^a^			AIFA ADRs n (%) ^a^			ARs or ADRs n (%) ^a^			Total %	
	Serious n = 49	Non-Serious n = 166	NR n = 1	Serious n = 283	Non-Serious n = 825	NR n = 51	ISS n = 216	AIFA n = 1159	*p* Value ^b^	on Ars ^c^	on Subjects ^d^
Gastrointestinal disorders	6 (12.2)	88 (53.0)	-	37 (13.1)	258 (31.3)	15 (29.4)	94 (43.5)	310 (26.7)	<0.001	29.4	52.5
Skin and subcutaneous tissue disorders	10 (20.4)	26 (15.7)	-	38 (13.4)	204 (24.7)	13 (25.5)	36 (16.7)	255 (22.0)	0.08	21.2	37.8
Nervous system disorders	2 (4.1)	7 (4.2)	-	38 (3.3)	85 (10.3)	7 (13.7)	9 (4.2)	130 (11.2)	0.002	10.1	18.1
Investigations	9 (18.4)	6 (3.6)	-	53 (18.7)	39 (4.7)	2 (3.9)	15 (6.9)	94 (8.1)	0.56	7.9	14.2
General disorders and administration site conditions	1 (2.0)	14 (8.4)	-	18 (6.4)	59 (7.2)	2 (3.9)	15 (6.9)	79 (6.8)	0.94	6.8	12.2
Psychiatric disorders	-	2 (1.2)	-	15 (5.3)	35 (4.2)	2 (3.9)	2 (0.9)	52 (4.5)	0.01	3.9	7.0
Musculoskeletal and connective tissue disorders	1 (2.0)	3 (1.8)	-	18 (6.4)	28 (3.4)	1 (2.0)	4 (1.9)	47 (4.1)	0.12	3.7	6.6
Renal and urinary disorders	6 (12.2)	5 (3.0)	-	16 (5.7)	19 (2.3)	1 (2.0)	11 (5.1)	36 (3.1)	0.14	3.4	6.1
Respiratory, thoracic and mediastinal disorders	4 (8.2)	1 (0.6)	-	8 (2.8)	22 (2.7)	-	5 (2.3)	30 (2.6)	0.81	2.5	4.5
Cardiac disorders	1 (2.0)	3 (1.8)	-	12 (4.2)	13 (1.6)	4 (7.8)	4 (1.9)	29 (2.5)	0.57	2.4	4.3
Vascular disorders	3 (6.1)	3 (1.8)	-	8 (2.8)	18 (2.2)	1 (2.0)	6 (2.8)	27 (2.3)	0.69	2.4	4.3
Metabolism and nutrition disorders	-	3 (1.8)	-	5 (1.8)	11 (1.3)	-	3 (1.4)	16 (1.4)	0.99	1.4	2.5
Eye disorders	-	-	-	1 (0.4)	12 (1.5)	2 (3.9)	-	15 (1.3)	0.09	1.1	1.9
Ear and labyrinth disorders	1 (2.0)	-	-	3 (1.1)	9 (1.1)	1 (2.0)	1 (0.5)	13 (1.1)	0.38	1.0	1.8
Immune system disorders	-	1 (0.6)	-	4 (1.4)	5 (0.6)	-	1 (0.5)	9 (0.8)	0.62	0.7	1.3
Hepatobiliary disorders	4 (8.2)	1 (0.6)	-	2 (0.7)	1 (0.1)	-	5 (2.3)	3 (0.3)	< 0.001	0.6	1.0
Reproductive system and breast disorders	-	1 (0.6)	-	1 (0.4)	5 (0.6)	-	1 (0.5)	6 (0.5)	0.92	0.5	0.9
Infections and infestations	-	-	-	2 (0.7)	2 (0.2)	-	-	4 (0.3)	0.39	0.3	0.5
Pregnancy. puerperium and perinatal conditions	-	1 (0.6)	1 (100.0)	1 (0.4)	-	-	2 (0.9)	1 (0.1)	0.01	0.2	0.4
Blood and lymphatic system disorders	1 (2.0)	-	-	2 (0.7)	-	-	1 (0.5)	2 (0.2)	0.40	0.2	0.4
Neoplasms benign, malignant and unspecified (incl cysts and polyps)	-	-	-	1 (0.4)	-	-	-	1 (0.1)	0.67	0.1	0.1
Not applied ^e^	-	1 (0.6)	-	-	-	-	1 (0.5)	-	-	0.1	0.1

AR: adverse reaction; ADR: adverse drug reaction; AIFA: Italian Medicines Agency; ISS: National Institute of Health. The total number of ARs exceeds the total number of subjects since a single patient report might include more than one suspected AR. ^a^ % refers to the total of each column. ^b^ Chi-square or Fisher’s exact tests were performed to compare ARs rate between ISS and AIFA. ^c^ Total % is calculated using the number of ARs belonging to the different SOCs as denominators: 216 from ISS + 1159 from AIFA = 1375. ^d^ Total % is calculated using the number of subjects reporting at least one AR as denominator: 127 from ISS + 643 from AIFA = 770. ^e^ AR/ADR not specified in the spontaneous report.

**Table 3:** Most frequently reported serious ARs and ADRs reports by SOCs and PTs.

SOCs and PTs	n = 332 (%)
**Investigations**	62 (18.7)
Hypercalcaemia	43 (12.9)
Hypervitaminosis D	4 (1.2)
Hypocalcemia	3 (0.9)
**Renal and urinary disease**	22 (6.6)
Renal failure	11 (3.3)
Nephrocalcinosis or kidney stones	4 (1.2)
Hydronephrosis	2 (0.6)
**Muscoloskeletal and connective disorders**	
Musculoskeletal pain	7 (2.1%)
Jaw osteonecrosis ^a^	6 (1.8%)
Others	5 (1.5%)
**Cardiac disorders**	13 (3.9)
Tachycardia (including cardiac arrhythmia and palpitations)	8 (2.4)
Chest pain	4 (1.2)
Myocardial infarction	1 (0.3)

AR: adverse reaction; ADR: adverse drug reaction. ^a^ Alendronate and cholecalciferol combination. Overall, causality assessment was “certain” in 8 cases, “probable/likely” in 298 cases, “possible” in 423 cases, “unlikely” in 9 cases and “unassessable/unclassifiable” in 32 cases (Table 1). As regards to serious cases, causality assessment was respectively “probable” and “possible” in 51 (29.6%) and 110 (63.9%) patients.

**Table 4:** Serious suspected ARs and ADRs according to demographic and clinical characteristics.

	Crude OR (95% CI)	Adjusted OR (95% CI)
**Age (years)**		
<30	1	1
30–65	1.48 (0.85–2.57)	0.76 (0.34–1.71)
≥65	1.46 (0.84–2.55)	0.75 (0.33–1.72)
**Sex**		
Male	1	1
Female	1.34 (0.91–1.97)	0.95 (0.54–1.70)
**Number of concomitant products**		
0	1	1
1–4	0.93 (0.63–1.38)	0.85 (0.51–1.42)
≥5	2.35 (1.41–3.94)	2.44 (1.30–4.60)
**Length of exposure (days)**		
≤7	1	1
8–30	1.70 (0.97–2.98)	2.08 (1.12–3.86)
>30	1.62 (1.03–2.53)	1.91 (1.16–3.15)
**VitDps reported daily dose (mg)**		
≤1000	1	-
>1000	2.70 (1.30–5.64)	-

AR: adverse reaction; ADR: adverse drug reaction.

## Data Availability

The data presented in this study are available in supplementary material and upon reasonable request from the corresponding author.

## Supplementary Materials

The following supporting information can be downloaded at: https://www.mdpi.com/article/10.3390/jcm12144726/s1, Table S1: Suspected adverse reactions associated with products containing Vitamin D reported to the Italian Phytovigilance system from February 2001 to August 2022.; Table S2: Suspected adverse reactions associated with products containing Vitamin D reported to the Italian Pharmacovigilance system from February 2001 to August 2022. Table S3: ARs distributions by System Organ Class (SOC) for each year pre-COVID-19 (2017–2018–2019). Table S4: ARs distributions by System Organ Class (SOC) for each year during the COVID-19 pandemic (2020–2021–2022). Table S5: Comparison ARs distributions by System Organ Class (SOC) between pre-COVID-19 (period 1: 2017–2019) and during the COVID-19 pandemic (period 2: 2020–2022). Table S6: ARs distributions by System Organ Class (SOC) for each year during the COVID-19 pandemic (2020–2021-2022).
