# Supplementary material for: Safety Profile of Vitamin D in Italy: An Analysis of Spontaneous Reports of Adverse Reactions Related to Drugs and Food Supplements
Source: J Clin Med. 2023 Jul 17;12(14):4726. doi: 10.3390/jcm12144726 (PMC10381134; doi:10.3390/jcm12144726)
Supplement: Supplementary file 1 [file jcm-12-04726-s001.zip › jcm-2407136-supplementary.pdf]

Supplementary Table 1. Suspected adverse reactions associated with products containing Vitamin D reported to the Italian Phytovigilance system from March 2002 to August 2022.

**Age (year)**	**Gender**	**Product[a] (Brand name)**	**Therapy length (day)**	**Time to onset[b] (day)**	**Adverse Reaction**	**Hospitalization**	**Recovery**	**Other Medications /Conditions**	**Dechallenge /Rechallenge**	**Causality assessment**
**6 months**	F	Divit D3®	NR	8	Vomiting	NR	Recovered/resolved	NR/NR	Positive/NR	Probable/likely
**NR**	F	Sustenium Multivitaminico 100®	NR	NR	Abdominal pain, Vomiting	NR	NR	NR/NR	NR/NR	Probable/likely
**45**	F	Nutrimamma Hp®	NR	NR	Stomach pain, vomiting	NR	Recovered/resolved	No/No	Positive/Positive	Probable/likely
**84**	M	Supradyn Ricarica®	2	3	Skin rash	NR	Recovered/resolved	NR/Ischemic stroke	NR/No due to product withdrawal	Probable/likely
**33**	F	Chirofert®	61	0	Urticarial vasculitis	NR	Recovered/resolved	NR/NR	Positive/Positive	Certain
**68**	M	Vitalife D®	444	444	Bone marrow aplasia	Yes	Recovering/resolving	Omega 3, Vitalife C®, Orac Spice®, Melatonin Complex, Multivitamineral/NR	Positive/NR	Possible
**29**	F	Multiup®	3	0	Headache, Nausea, Vomiting,	NR	Recovered/resolved	Indoxen®/ Chronic headache	Positive/Positive	Probable/likely
**54**	M	Herbalife Formula 1®	34	34	Colestatic epatitis	NR	NR	NR/NR	NR/No due to product withdrawal	Probable/likely
**39**	F	Vitamin D E A	21	21	Colestatic epatitis	Life threatening	Persistent reaction	Tachipirina®, Gan Mao Influ®, Fu Fang pills, Vitaderm Leucand®, Levoxacin®, Minulet®/ Pyrexia, respiratory tract inflammation	NR/No due to product withdrawal	Possible

**18**	F	Sinua®	29	31	Autoantibody-positive test, acute renal block, hypocalcemia, hypoproteinemia	Yes	Recovering/resolving	Diane®, Flexiban®/NR	Positive/No due to product withdrawal	Probable/likely
**34**	F	Merluzzovis®	NR	NR	Renal failure, hypercalcemia, nephrocalcinosis	Yes	NR	Broncovalea®, Intolvis®, Liquirvis®, Carota-T, Ecoval®, Plasil®/Celiac disease, grasses and cat hair allergy, asthma	NR/No due to product withdrawal	Certain
**15 days**	M	Ditrevit K®	NR	NR	Breath-holding episodes	Life threatening	Recovered/resolved	NR/NR	NR/NR	Possible
**78**	M	Multivitamin complex	NR	NR	Palpitation	Yes	Recovered/resolved	Guaranà Herbalife, Omega3, Proteic dietary supplement, Nutritional Shake Milk/NR	Positive/No due to product withdrawal	NR
**57**	M	Merluzzovis®	306	343	Acute renal failure, hypercalcemia, hypervitaminosis D, nephrotoxicity, ear ringing	Yes	Recovered/resolved	Antihypertensives/Anxiety, hypertension	NR/No due to product withdrawal	Certain
**59**	F	Galenic preparation (Calcium, Vitamin D)	26	27	Renal failure, hypercalcemia	Yes	NR	Lansoprazole, NSAIDs/NR	NR/No due to product withdrawal	Probable/likely
**82**	M	Massigen®	12	12	INR increase	NR	Recovered/resolved	Cardiofenone®, Casodex®, Citalopram®, Coumadin®, Finastid®, Lacirex®/NR	NR/No due to product withdrawal	Probable/likely
**25**	F	Multicentrum®	NR	3	Nausea, esophageal reflux, facial and mouth pruritus, malaise	NR	Recovered/resolved	Lexotan®/NR	Positive/No due to product withdrawal	Probable/likely
**55**	F	Fitormil Omega®	34	28	Facial oedema, desquamation	NR	NR	Symbicort®/NSAIDs sensitivity	NR/No due to product withdrawal	Possible
**42**	F	Neomam®	NR	NR	Miscarriage	NR	NR	Sideromap®/Pregnacy	NA/NA	Unlikely

**2 months**	M	Ditreol D3 Dha®	2	2	Bronchospasm	Yes	Recovered/resolved	NR/NR	Positive/No due to product withdrawal	Possible
**1 month**	M	Ditrevit Forte®	NR	0	Breathing problems	NR	Recovered/resolved	NR/NR	NR/No due to product withdrawal	Possible
**64**	M	Supradyn Vital Age 50®	1	2	INR decrease	NR	Recovered/resolved	Ramipril®, Norvasc®, Carvedilol, Propafenone, Lansoprazole, Sintrom®/NR	NR/No due to product withdrawal	Probable/likely
**56**	F	K2Vas®	11	9	Arrhythmia	NR	Recovered/resolved	Blopress®, Crestor®, Dibase®/Drug sensitivity	Positive/Positive	Certain
**57**	F	Ensure Plus Advance®	NR	NR	Diarrhoea	NR	Recovered/resolved	Bisoprolol, Cardirene®, Eutirox®/NR	Positive/Positive	Certain
**30**	F	Multicentrum Materna®	5	3	Erythema, pruritus	Yes	Recovering/resolving	NR/NR	NR/No due to product withdrawal	Probable/likely
**79**	M	Reumadep®	NR	NR	Diarrhoea, dehydration, mouth ulcers	NR	Recovered/resolved	NR/NR	Positive/No due to product withdrawal	Probable/likely
**75**	F	Esterol®	NR	1	Retrosternal burning	NR	Recovered/resolved	Bisoprolol, Cordarone®, Coumadin®, Eutirox®, Inspra®, Ramipril®/NR	NR/No due to product withdrawal	Probable/likely
**73**	F	Reumadep®	2	3	INR increase	NR	Recovered/resolved	Sintrom®, Sotalex®, Lasix®/NR	NR/No due to product withdrawal	Probable/likely
**24**	F	Vitamineral®	NR	0	Stevens Johnson syndrome	Yes	NR	Ciproxin®, Deltacortene®/NR	NR/No due to product withdrawal	Possible
**31**	F	Multivit®	6	6	Stomach acidity, urticaria, nausea	NR	Recovered/resolved	NR/NR	Positive/No due to product withdrawal	Probable/likely
**3**	F	Apetit®	8	8	Skin rash, pruritus	NR	Recovered/resolved	Veclam®/NR	Positive/NR	Possible
**NR**	F	Polase Ricarica Inverno®	NR	NR	Blood pressure increase, fear	NR	NR	NR/Hypertension	NR/No due to product withdrawal	Possible

**52**	M	Multicentrum Uomo 50+®	NR	NR	Dysuria, fever, urinary frequency alteration, incontinence	NR	NR	NR/NR	NR/No due to product withdrawal	Possible
**44**	F	Slimquick Pure®	NR	NR	Acute epatitis	Yes	NR	NR/Obesity	NR/No due to product withdrawal	Probable/likely
**72**	F	Cartijoint D®	1	1	Lip swelling, dry mouth	NR	Recovered/resolved	NR/NR	Positive/No due to product withdrawal	Probable/likely
**NR**	M	Multicentrum Uomo®	NR	14	Malaise	NR	NR	NR/NR	NR/No due to product withdrawal	Possible
**43**	F	Ensure Plus®	NR	384	Diarrhoea, swelling, abdominal pain	NR	Recovered/resolved	NR/NR	Positive/Positive	Certain
**24**	F	Natalben®	NR	0	Abnormal uterine contractions	NR	NR	Buscopan®, Vitamin B complex/Pregnancy	NR/No due to product withdrawal	Possible
**23**	M	Psor®	NR	NR	Eyelid edema, fever, pruritus	NR	Recovered/resolved	Oki®/Psoriasis	NR/No due to product withdrawal	Possible
**58**	F	Bariatric®	4	0	Oedema of the lips, urticarial rash	Yes	Recovered/resolved	Aponil®, Appryo®, Eutirox®, Ivor®, Lastan®/NR	NR/No due to product withdrawal	Possible
**45**	F	Climavera®	9	44	Nipple pain	NR	Recovered/resolved	Promensil Forte®/NR	Positive/Negative	Possible
**70**	F	Curcudyn®	31	31	Psoriasiform rash	NR	Recovered/resolved	Allopurinol, Carvedilol, Clopidogrel, Lasix®/Diabetes	Positive/No due to product withdrawal	Possible
**49**	M	Multicentrum®	NR	0	Urticaria	Yes	Recovering/resolving	Avodart®/Food allergy	NR/No due to product withdrawal	Possible
**49**	F	Inneov Densilogy Alpha®	2	0	Headache, abdominal pain, spontaneous hematoma	NR	NR	NR/NR	NR/No due to product withdrawal	Probable/likely
**37**	F	Inneov Densilogy®	44	0	Digestive problems	NR	NR	NR/NR	NR/No due to product withdrawal	Probable/likely

**37**	F	Inneov Densilogy®	44	42	Diarrhoea	NR	NR	NR/NR	NR/No due to product withdrawal	Probable/likely
**12 days**	M	Xinovit®	NR	NR	Alterated mood	NR	Recovered/resolved	NR/NR	Positive/No due to product withdrawal	Probable/likely
**NR**	NR	Multicentrum Uomo 50+®	NR	0	Dark urine	NR	NR	NR/NR	NR/No due to product withdrawal	Probable/likely
**44**	F	Multicentrum Donna®	NR	0	Reaction to drug eccipient	NR	NR	NR/Lactose intolerance	NR/No due to product withdrawal	Probable/likely
**69**	F	Multicentrum Donna 50+®	2	0	Vomiting	NR	Recovered/resolved	NR/ Valvular heart disease	NR/No due to product withdrawal	Probable/likely
**NR**	NR	Swisso Uomo 50+®	NR	NR	Chromaturia	NR	NR	NR/NR	NR/No due to product withdrawal	Possible
**2**	F	Multicentrum Junior®	NR	0	Abuse, abdominal pain	NR	Recovering/resolving	NR/NR	Positive/No due to product withdrawal	Probable/likely
**40**	F	Inneov Densilogy Alpha®	2	1	Ocular edema	NR	NR	NR/NR	NR/No due to product withdrawal	Probable/likely
**45**	F	Inneov Densilogy Alpha®	1	2	Ocular edema	NR	NR	NR/NR	NR/NR	Probable/likely
**49**	F	Estromineral®	NR	0	Urticaria	Yes	Recovered/resolved	NR/Allergies	Positive/No due to product withdrawal	Probable/likely
**NR**	M	Novalac1®	153	153	Dysentery	NR	Recovered/resolved	NR/NR	Positive/No due to product withdrawal	Probable/likely
**75**	F	Leniart Uc-Ii®	30	0	Headache	NR	Recovering/resolving	Mepral®, Triatec®, Toradiur®, Dibase®, Clasteon®/NR	Positive/No due to product withdrawal	Possible
**44**	F	Ganoderma Lucidum, Cordyceps sinensis	98	98	Estrogen increase, FSH decrease	NR	Persistent reaction	No/No	NR/No due to product withdrawal	Probable/likely

**31**	F	Natalben Piu'®	NR	NR	Allergic reaction	NR	Recovered/resolved	NR/NR	NR/No due to product withdrawal	Possible
**46**	M	Deflogo Junior®	NR	0	Fever, gastroesophageal reflux, vomiting	Yes	Recovered/resolved	Rinazina®/Smoker	Positive/No due to product withdrawal	Probable/likely
**61**	F	Integral T®	NR	NR	Cramps, myalgia, heaviness	NR	Recovered with sequelae	Diserinal®, Eutirox®, Mederos®/NR	Positive/No due to product withdrawal	Possible
**2 months**	F	Novalac Reflux®	NR	3	Diarrhoea	NR	NR	NR/NR	NR/No due to product withdrawal	Probable/likely
**48**	F	Prolei Complex®, Rock D3®	13	2	Bone pain, dizziness, parathyroid hormone plasmatic level alteration	Other condition	NR	Prolia®, Femara®/Dizziness	NR/NR	Possible
**22 months**	M	Guna Vitformula®	0	0	Urticaria	NR	Recovered/resolved	NR/NR	NR/No due to product withdrawal	Probable/likely
**52**	F	Swisse Donna 50+®	NR	2	Multiple sclerosis worsening	NR	NR	NR/Spastic sclerosis	NR/No due to product withdrawal	Possible
**32**	F	Multicentrum Mamma®	5	364	Nausea, vomiting	Other condition	Recovered/resolved	No/No	NR/No due to product withdrawal	Possible
**79**	F	Supradyn Ricarica®	3	2	Nausea, vomiting	NR	Recovered/resolved	Cardioaspirin®, Antihypertensives/NR	Positive/No due to product withdrawal	Probable/likely
**42**	F	Supradyn Ricarica®	6	5	Diarrhoea, nausea	NR	Recovered/resolved	NR/NR	Positive/No due to product withdrawal	Probable/likely
**53**	F	Supradyn Ricarica 50+®	2	0	Diaphoresis	NR	Recovered/resolved	NR/NR	Positive/No due to product withdrawal	Probable/likely
**54**	F	Vitamin D Metagenics®	NR	NR	Stomach pain	NR	Recovered/resolved	Eutirox®, Libradin®/NR	Positive/No due to product withdrawal	Probable/likely

**20**	M	Solgar Supplement Vm-2000®	5	1	General pruritus, local rash	NR	Persistent reaction	NR/Allergy not specified	Negative/No due to product withdrawal	Possible
**80**	F	Nutrison®	NR	0	Oedema of the lips	NR	Recovered/resolved	Ceftriaxone, Levofloxacine/NR	Positive/No due to product withdrawal	Probable/likely
**18**	F	IperAla®	NR	NR	Urticarial rash	NR	NR	NR/NR	NR/NR	Possible
**4 days**	F	Ditrevit Forte®	NR	0	Stomach cramps, abnormal stools, meteorism, uncontrollable crying	NR	Recovered/resolved	NR/NR	Positive/No due to product withdrawal	Probable/likely
**45 days**	F	Novalac Reflux®	NR	1	Dysentery	NR	NR	NR/NR	NR/No due to product withdrawal	Probable/likely
**90**	F	Ensure Advance Plus®	NR	NR	Subtherapeutic INR	Life threatening	NR	Coumadin®/NR	NR/No due to product withdrawal	Possible
**50**	M	Heliocare Ultra D®	0	21	Bleeding disorder, epistaxis, reduced clotting factors	Yes	Recovering/resolving	NR/NR	Positive/No due to product withdrawal	Probable/likely
**41**	F	Normotir®	NR	NR	Hyperpyrexia	NR	NR	NR/NR	NR/NR	Possible
**71**	F	Resource Repair®	3	2	Digestive problems, nausea, stomach heaviness	NR	Recovered/resolved	Acetylsalicylic acid, Aldactone®, Amiodarone, Cardicor®, Corlentor®, Esomeprazole, Folidar®, Tardyfer®, Apidra SoloStar®, Binocrit®, Clexane®, Plasbumin®, Lasix®, Isoram®/NR	NR/No due to product withdrawal	Probable/likely
**78**	M	Resource Repair®	3	2	Diarrhoea	NR	Recovered/resolved	Allopurinol, Amiodarone, Potassium canrenoate, Cardicor®, Deursil®, Furosemide, Pantoprazole, Torvast®, Ambroxol Dorom®, Clenil®, Coumadin®/NR	NR/No due to product withdrawal	Probable/likely

**62**	F	Ymea Vitality®	567	567	Hepatitis	Yes	Recovering/resolving	NR/Hepatic cytonecrosis modification	Positive/No due to product withdrawal	Probable/likely
**5 months**	F	Vitamin D3 Bonavita	2	2	Appetite lack, vomiting	NR	Recovered/resolved	NR/NR	NR/NR	Probable/likely
**34**	F	Sustenium Bioritmo3®	5	0	Abnormal hunger, hyperhidrosis, tachycardia	NR	Recovered/resolved	NR/NR	Positive/No due to product withdrawal	Probable/likely
**68**	F	Eumetab®	0	0	Vomiting, diarrhoea	NR	Recovered with sequelae	Omega Formula Della Guna/NR	Positive/Positive	Probable/likely
**2 months**	M	Ditrevit Forte®	8	4	Colic	NR	Recovering/resolving	NR/NR	Positive/Positive	Certain
**5 months**	M	Idroplurivit D+®	122	122	Appetite lack	NR	Recovered/resolved	No/No	Positive/Positive	Certain
**7 months**	M	Novalac®, Proton®	2	0	Diarrhoea	NR	Recovered/resolved	No/No	NR/NR	Probable/likely
**38**	F	Sustenium Bioritmo3®	NR	302	Hot flashes, nausea, vomiting	NR	Recovered/resolved	NR/NR	No due to continuation of treatment	Possible
**8 months**	F	Novalac®, Vitamin D3 Bonavita	NR	NR	Vomiting, constipation	NR	Persistent reaction	Mylicon®/NR	NR/NR	NR
**4 months**	NR	Novalac®, Dicovit D®	NR	4	Gastrointestinal pain, constipation, vomiting	NR	Recovered/resolved	Mylicon®/Intestinal colics	NR/NR	Probable/likely
**7 months**	M	Ditrevit®	NR	NR	Breathing problems, dyspnea	Yes	Recovering/resolving	NR/NR	Positive/NR	NR
**1 months**	M	Novalac®, Ditrevit Forte®	NR	NR	Diarrhoea	NR	Recovered/resolved	NR/NR	NR/NR	NR
**3 months**	M	Novalac®,Vitamin D Nos	NR	NR	Diarrhoea	NR	NR	NR/NR	NR/NR	NR

**15 days**	F	Novalac Reflux Pro®, Ditrevit Forte®	NR	NR	Skin rash, vomiting	NR	Recovered/resolved	NR/NR	Positive/No due to product withdrawal	NR
**34**	F	Multicentrum Mamma®	19	19	Skin pruritus	NR	Recovered/resolved	NR/ Autoimmune thyroiditis	Positive/No due to product withdrawal	Probable/likely
**NR**	NR	Novalac Allernova Pro®	4	0	Vomiting	NR	NR	NR/NR	NR/No due to product withdrawal	Probable/likely
**57**	F	Flexart®	60	59	Palpable liver	Invalidating	Persistent reaction	Levotiroxine/Hypothyroidism, cholesterolemia, glaucoma, lumbosciatalgia, tension headache, anemia, lactose intolerance, periarthritis and calcifications, cervical and lumbar osteoarthritis, rhizoarthritis, seborrheic dermatitis	No due to continuation of treatment	Possible
**72**	F	Vitamin D	NR	0	Bitter taste	NR	Persistent reaction	NR/NR	NR/Positive	Probable/likely
**39**	F	Nutrylife Vit®	14	14	Guillain-Barré syndrome	Yes	NR	NR/NR	NR/NR	Possible
**3 months**	F	Novalac Reflux Pro®, Ditrevit Forte®	NR	NR	Aerophagia, gastroesophageal reflux, soft stool, moaning	NR	NR	Lucen®/ Gastroesophageal reflux	NR/NR	NR
**48 day**	F	Novalac As1®	NR	NR	Vomiting, fever, rash	NR	NR	No/No	NR/NR	NR
**5 months**	M	Novalac Reflux®, Ditrevit Forte®,	NR	NR	Aerophagia, vomiting, abnormal crying	No	Persistent reaction	Carvedilol/Gastroesophageal reflux	Negative/No due to product withdrawal	NR
**NR**	F	Novalac Allernova®	NR	NR	NR	NR	NR	NR/NR	NR/NR	NR
**62**	F	RV+ Immuno®	6	6	Erythema	NR	Recovered/resolved	NR/Ragweed and cypress allergy	Positive/NR	Possible
**36**	F	Chirofert Plus®	NR	NR	Headache, gastroesophageal reflux, gastritis	NR	Recovered/resolved	NR/NR	Positive/Positive	Probable/likely

**5 months**	M	Novalac Reflux Pro®, Vitamin D3, Lutein D3	NR	NR	Black stool	NR	NR	NR/NR	NR/NR	NR
**5 months**	M	Novalac®, Vitamin D Nos	30	NR	Diarrhoea	NR	NR	NR/NR	NR/NR	Possible
**59**	M	Evocolest®	NR	NR	Nausea, vomiting, diarrhoea	NR	NR	NR/NR	NR/Positive	Probable/likely
**NR**	M	Vitamin D3	NR	NR	Esophageal reflux	NR	Persistent reaction	NR/Hiatal hernia	No due to continuation of treatment	Probable/likely
**51**	F	Curcudyn Forte®	21	0	Malaise, hypertransaminasemia	NR	Recovered/resolved	NR/NR	Positive/No due to product withdrawal	Probable/likely
**NR**	M	Vitamin A, B6, B9, B12, C, D, Zinc, Copper, Iron, Selenium, Magnesium (Mag immuno plus®)	NR	NR	Vomiting, gastrointestinal malaise	NR	NR	NR/NR	NR/NR	Possible
**22**	F	Swisse Ragazza®	NR	NR	Nausea, vomiting	Invalidating	Persistent reaction	NR/NR	NR/NR	NR
**NR**	F	Vitamin A, B6, B9, B12, C, D, Zinc, Copper, Iron, Selenium, Magnesium (Mag immuno plus®)	NR	NR	Nausea	NR	NR	NR/NR	NR/NR	NR
**NR**	F	Vitamin A, B6, B9, B12, C, D, Zinc, Copper, Iron, Selenium, Magnesium (Mag immuno plus®)	NR	NR	Nausea, headache	NR	NR	NR/NR	NR/NR	NR
**47**	F	Vitamin A, B6, B9, B12, C, D, Zinc, Copper,	NR	NR	Nausea, vomiting	NR	NR	NR/NR	NR/NR	NR

		Iron, Selenium, Magnesium (Mag immuno plus®)								
**NR**	F	Vitamin A, B6, B9, B12, C, D, Zinc, Copper, Iron, Selenium, Magnesium (Mag immuno plus®)	NR	NR	Vomiting	NR	NR	NR/NR	NR/NR	NR
**4 months**	M	Neolatte 1®	NR	NR	Vomiting, swelling, skin rash, slowness, apathy	NR	Recovered/resolved	NR/NR	NR/NR	NR
**2 months**	F	Novalac Reflux Pro®	NR	NR	Hard stool	NR	NR	NR/NR	NR/NR	NR
**NR**	M	Vitamin A, B6, B9, B12, C, D, Zinc, Copper, Iron, Selenium, Magnesium (Mag immuno plus®)	NR	NR	Nausea	NR	Recovered/resolved	NR/NR	NR/NR	NR
**NR**	F	Vitamin A, B6, B9, B12, C, D, Zinc, Copper, Iron, Selenium, Magnesium (Mag immuno plus®)	NR	NR	Nausea	NR	NR	NR/NR	NR/NR	NR
**NR**	F	Vitamin A, B6, B9, B12, C, D, Zinc, Copper, Iron, Selenium, Magnesium (Mag immuno plus®)	NR	NR	Nausea, vomiting	NR	NR	NR/NR	NR/NR	NR
**38**	F	Kallergen D®	NR	NR	Abdominal bloating	NR	NR/NR	NR	NR/NR	NR

**NR**	NR	Vitamin A, B6, B9, B12, C, D, Zinc, Copper, Iron, Selenium, Magnesium (Mag immuno plus®)	NR	NR	Nausea, vomiting	NR	NR	NR/NR	NR/NR	NR
**NR**	F	Vitamin A, B6, B9, B12, C, D, Zinc, Copper, Iron, Selenium, Magnesium (Mag immuno plus®)	NR	NR	Abdominal pain, nausea	NR	NR	NR/NR	NR/NR	NR
**NR**	F	Vitamin A, B6, B9, B12, C, D, Zinc, Copper, Iron, Selenium, Magnesium (Mag immuno plus®)	NR	NR	Nausea, vomiting	NR	NR	NR/NR	NR/Positive	NR
**7**	F	Vitamin D3	19	16	Abdominal pain, accidental abuse	NR	Recovering/resolving	NR/NR	NR/NR	Probable/likely
**63**	F	Immunactive Pharcos®	1	1	Pruritus, burning skin, rash	Yes	Recovering/resolving	Folic acid, liposomal Curcuma longa/Antibiotic allergy	NR/NR	Possible

NR: not reported

Supplementary Table 2. Suspected adverse drug reactions associated with products containing Vitamin D reported to the Italian Pharmacovigilance system from March 2002 to August 2022.

Age (Year)	Gender	Medicinal Product (brand name)	Duration Of Treatment (Day)	Time to onset [b] (Day)	Adverse Drug Reaction	Seriousness	Recovery	Other Medications	Dechallenge	Rechallenge	Causality Assessment
76	F	CACIT®	NR	NR	Dyspepsia, Nausea	No	Recovered/resolved	NORVASC®	Positive	NR	Possible
71	F	ATITEN®	NR	NR	Fever	No	Recovered/resolved	EUTIROX®, DEURSIL®	NR	NR	Unlikely
45	M	CALCIJEX®	NR	NR	Chest Pain, Tachycardia	Yes	Recovered/resolved	EPOETINA BETA	NR	NR	Possible
70	M	CALCIJEX®	NR	NR	Chest Pain, Dyspnea, Tachycardia	Yes	Recovered/resolved	BENADON®	NR	NR	Possible
25	F	CALCIJEX®	NR	NR	Chest Pain, Dyspnea, Tachycardia	Yes	Recovered/resolved	EPOETINA BETA	NR	NR	Possible
76	F	FOSCALD3®, ARTILOG®	NR	NR	Acute Urticaria	Yes	NR	NR	NR	NR	Possible
71	F	OSTEOFOSD3®	NR	NR	Abdominal Pain, Vomiting	No	Recovered/resolved	NR	Positive	NR	Probable/likely
62	F	CACIT®	NR	NR	Cough, Dyspepsia	Yes	NR	EOLUS®	NR	NR	Possible
63	F	CACIT ®, DIFOSFONAL®	NR	NR	Gastroesophageal Reflux, Nausea	Yes	Recovered/resolved	NR	NR	NR	Possible
65	F	CACIT ®, DIFOSFONAL®	227	227	Abdominal Pain	Yes	NR	TAVOR®	NR	NR	Possible
65	F	CACIT ®, DIFOSFONAL®	273	273	Abdominal Pain	Yes	NR	TAVOR®	NR	NR	Possible
NR	F	OSTEOFOSD3®	NR	NR	Cutaneous Rash	NR	NR	CONTROL®	NR	NR	Possible
45	F	ROCALTROL®	NR	NR	Constipation, Headache, Nausea, Somnolence	NR	Recovered/resolved	NR	NR	NR	Probable/likely
67	F	CACIT®, DIFOSFONAL®	NR	NR	Hepatitis	Yes	NR	NR	NR	NR	Unlikely
67	F	CACIT®, DIFOSFONAL®	NR	NR	Abdominal Pain, Nausea	Yes	Recovered/resolved	NR	NR	NR	Possible
90	M	CALCIUM D3 SANDOZ®	NR	NR	Cutaneous Rash	Yes	Persistent	NR	NR	NR	Probable/likely
54	F	CALCIUM D3 SANDOZ®	0	0	Tachycardia	NR	Recovered/resolved	FEMIPRES PLUS®, XARATOR®, CARDIOASPIRIN®	Positive	NR	Possible

72	F	EUROCAL D3®	NR	NR	Diarrhoea	NR	Recovered/resolved	EUTIROX®, DELTACORTENE®, LEXOTAN®, HALCION®	NR	NR	Possible
73	F	CACIT®	NR	NR	Gastrointestinal Pain	No	NR	NAPRILENE®	NR	NR	Possible
68	F	OSTEOFOSD3®	10	11	Erythema	No	NR	DELTACORTENE®	NR	NR	Possible
82	F	ROCALTROL®	25	28	Urticaria	NR	Recovered/resolved	TICLOPIDINA	Positive	NR	Possible
53	F	OSTEOFOSD3®	NR	NR	Abdominal Pain	NR	Recovered/resolved	MODURETIC®	NR	NR	Possible
70	M	DEDIOL®	15	15	Hypercalcaemia	NR	NR	GENALEN®	NR	NR	Possible
61	F	DIFIX®	NR	NR	Serum Cholesterol Increased	NR	NR	NR	NR	NR	Possible
67	F	DEDIOL	13	13	Agitation, Tachycardia, Rash	NR	Recovered with sequelae	LOPRESOR®, MODURETIC®, ENAPREN®, PARIET®, SINVACOR®	Positive	NR	Possible
48	F	NATECAL D3®	6	6	Concentration And Memory Impaired, Logorrhoea, Hearing Impairement	No	Recovered/resolved	NR	Positive	NR	Probable/likely
NR	F	OSTEOFOSD3®	NR	NR	Pruritus, Exantema	NR	Recovered/resolved	NR	Positive	NR	Probable/likely
80	F	NATECAL D3®	17	17	Epigastric Pain	NR	NR	TAVOR®, OPTRUMA®	NR	NR	Possible
59	F	OSTEOFOSD3®	0	61	Tachycardia	NR	Recovered/resolved	NR	Positive	NR	Probable/likely
57	F	CACIT®	77	85	Diarrhoea	No	Recovered/resolved	NR	Positive	NR	Probable/likely
74	F	NATECAL D3®	55	56	Gingivitis	NR	Recovered/resolved	CLODY®	NR	NR	Possible

66	F	OSTRAM D3®	294	294	Erythema	Yes	Recovered/resol<br>ved	UNIPRILDIUR®	Positive	NR	Possible
55	F	EUROCAL D3®	8	8	Angioedema	NR	Recovered/resol<br>ved	NR	Positive	NR	Probable/li<br>kely
68	F	CACIT®	7	7	Dermatitis	NR	Recovered/resol<br>ved	LASIX®,<br>KANRENOL®,<br>PORTOLAC®,<br>PANTECTA®,<br>KONAKION®,<br>ALENDRONAT<br>O SODICO	Positive	NR	Possible
56	F	OSTEOFOSD3®	47	45	Dyspepsia,<br>Pyrosis	No	Recovered/resol<br>ved	NR	Positive	NR	Probable/li<br>kely
71	F	OSTRAM D3®	0	1	Pruritus, Rash	No	Recovered/resol<br>ved	NR	Positive	NR	Probable/li<br>kely
80	F	OSTEOFOSD3®	NR	10	Epigastric Pain,<br>Dyspepsia	No	NR	DIFOSFONAL®	NR	NR	Possible
50	F	OROTRE®	15	15	Gastritis	NR	NR	ALENDROS®	NR	NR	Possible
82	F	OSTRAM D3®	30	0	Pirosis	NR	Recovered/resol<br>ved	SEREVENT®,<br>OXIVENT®,<br>CLENIL®	Positive	NR	Possible
70	F	METOCAL®	8	6	Abdominal Pain	No	Recovered/resol<br>ved	NR	Positive	NR	Probable/li<br>kely
40	F	METOCAL®	9	7	Abdominal Pain	No	Recovered/resol<br>ved	NR	Positive	NR	Probable/li<br>kely
55	F	CALCIUM D3 SANDOZ®	4	32	Dermatitis	NR	Recovered/resol<br>ved	FOSAMAX®	NR	NR	Unlikely
80	F	CALCICOL D3®,<br>ECAFAST®, REQUIP®	8	8	Visual<br>Hallucinations	NR	Recovered/resol<br>ved	MODURETIC®,<br>NAPRILENE®,<br>FLEBIL®	Positive	NR	Possible
59	F	EUROCAL D3®, CALCIUM<br>D3 SANDOZ®	0	108	Confusional<br>State, Headache,<br>Hyperidrosis,<br>Malaise,<br>Tachycardia	NR	Recovered/resol<br>ved	NR	Positive	NR	Possible
63	F	EUROCAL D3®, VIOXX®,<br>ACTONEL®	9	9	Urticaria	NR	NR	NR	NR	NR	Possible
47	F	DIFIX®	1	0	Confusion,<br>Headache,	No	Recovered/resol<br>ved	ELOCON®	Positive	NR	Possible

					Nausea, Mucosal Tingling						
65	F	EUROCAL D3®	11	0	Diarrhoea, Mumbling	NR	Recovered/resolved	NR	Positive	NR	Probable/likely
76	F	NATECAL D3®, NIMESULIDE	2	1	Diplopia	Yes	Persistent	EUTIROX®, TRITTICO®, PERSANTIN®, PANTECTA®, DEDIOL®	Negative	NR	Possible
66	F	CACIT®	7	3	Pruritus, Rash	No	NR	PROTAPHANE®, EUTIROX®, LOBIVON®, RATACAND®	NR	NR	Possible
70	F	NATECAL D3®, ADRONAT®	21	20	Vasculitis	Yes	Persistent	LUCEN®, MOTILIUM®, ASCRIPTIN®	NR	NR	Possible
82	F	CALPLUSD3®, ZYLORIC®	54	51	Agranulocytosis, Dermatitis Exfoliative, Mucositis NOS, Oral Moniliasis, Bronchopneumonia	Yes	Fatal	APROVEL®®, CATAPRESAN TTS®®, CARDIOASPIRIN®®, SIVASTIN®	Negative	NR	Possible
59	F	EUROCAL D3®	1227	1218	Epigastric Pain	No	Recovered/resolved	NR	Positive	NR	Probable/likely
57	M	CACIT ®, DRONAL®	1368	1368	Urticaria	No	NR	NR	NR	NR	Possible
76	F	OSTELIN®, INFLEXAL V®	0	0	Urticaria	No	Persistent	LEXOTAN®, LENDORMIN®, BETOPTIC®, COMBISARTAN®, GLICONORM®, TAPAZOLE®, BEZALIP®, EDRONAX®, TICLOPIDINA	Negative	NR	Possible
56	F	CACIT®	2	2	Acute Urticaria	No	Recovered/resolved	NR	Positive	NR	Probable/likely

72	F	EUROCAL D3®, OSTEOFOSD3®	4	5	Gastritis	No	Recovered/resolved	COMBISARTAN®	Positive	NR	Possible
72	F	EUROCAL D3®, OSTEOFOSD3®	4	4	Gastritis	No	Recovered/resolved		Positive	NR	Possible
46	M	DISEON®	0	0	Headache (Migraine)	No	Recovered/resolved	DIFLUCAN®	Positive	NR	Possible
65	F	COLECALCIFEROLO	7	7	Oppression	NR	Recovered/resolved	NR	Positive	Positive	Probable/likely
69	F	DISEON®	1081	1076	Conjunctiva Affection	No	Recovered/resolved	RATACAND PLUS®, CONCOR®, ZANEDIP®	Positive	NR	Possible
56	F	CALCIO CARBONATO + VITAMINA D3 RATIOPHARM®	4	4	Retrosternal Pain, Dysphagia	No	Recovered/resolved	SYSTEM®, PASADEN®	Positive	NR	Possible
80	F	CACIT®	0	0	Erythematous Skin Rash	Yes	Recovered/resolved	NR	Positive	NR	Probable/likely
85	F	CACIT®	3	2	Dermatitis	No	NR	LANOXIN®, LASIX®, SPIROFUR®, ENAPREN®, CARVEDILOLO	NR	NR	Possible
66	F	BIOCALCIUM D3®	2	0	Visual Impairment	No	Recovered/resolved	ISOPTIN®, CARDIRENE®	Positive	NR	Possible
82	F	CALCITRIOLO TEVA ®, ALLOPURINOLO TEVA	3	3	Dermatitis, Hyperpyrexia, Generalized Pruritus	Yes	Persistent	HUMULIN®, LASIX®, ISMO®, ZANEDIP®, NEURONTIN®, CABASER®, MINITRAN®, LOPRESOR®, RIVOTRIL®, STUGERON®, NEORECORMON®, TICLOPIDINA CLORIDRATO	Positive	NR	Possible
63	F	ROCALTROL®	88	88	Constipation	No	Recovered/resolved	NIKLOD®	Positive	NR	Possible

32	M	SILKIS®	41	41	Burning Infusion Site, Facial Redness	No	Recovered/resolved	NR	Positive	NR	Probable/likely
78	F	METOCAL®	7	5	Epigastralgia	No	Recovered/resolved	UNIPRIL®	Positive	NR	Possible
70	F	ROCALTROL®	9	6	Erythema, Pruritus	No	Recovered/resolved	OPTINATE®	Positive	NR	Possible
59	F	CACIT®	NR	1398	Hematemesis (Gastric Ulceration)	Yes	NR	SINECOD TOSSE®	NR	NR	Possible
60	F	NATECAL D3®	187	71	Gingivitis, Stomatitis	No	Recovered with sequelae	LIVIAL®	Positive	NR	Possible
51	F	NATECAL D3®	193	193	Constipation	No	Recovered/resolved	NR	Positive	NR	Probable/likely
76	F	CALPLUSD3®	108	108	Constipation	No	Recovered/resolved	IGROTON®, CARDIOASPIRIN®, VERAPAMIL MYLAN GENERICS®, NAPRILENE®	Positive	NR	Possible
52	F	NATECAL D3®	NR	30	Gastric Pain	No	Recovered/resolved	NAPRILENE®, ESIDREX®, HALCION®, GAVISCON ADVANCE®	Positive	NR	Possible
53	F	CACIT®	41	41	Measles-Like Rash	No	NR	NR	NR	NR	Probable/likely
77	F	CALCIO CARBONATO + VITAMINA D3 GERMED®	28	17	Abdominal Pain, Diarrhoea	No	Recovered/resolved	DICLOREUM®, LIMPIDEX®, PERSANTIN®, VERAPAMIL BIG®	Positive	Positive	Probable/likely
52	F	CALCIO CARBONATO VITAMINA D3 EG®	45	4	Diarrhoea	No	Recovered/resolved	NR	Positive	NR	Probable/likely
68	M	DIBASE®, AGRIPPAL S1®	0	4	Arthralgia, Asthenia, Hypotension	No	Recovered/resolved	CARDIOASPIRIN®, DILATREND®, GLIBEN F®, TRIATEC®,	Positive	NR	Possible

								AVODART®, XATRAL®, STILNOX®, AMIODARONE			
69	F	CACIT®, ACTONEL®, ROCALTROL®	2	0	Urticaria	No	Recovering/resolving	NR	Positive	NR	Possible
58	F	DIFIX®, EUTIROX®	293	294	Presyncope	No	Recovered/resolved	NR	Positive	NR	Possible
53	F	DIBASE®, NATECAL D3®	137	32	Alopecia	No	NR	TAMOXIFENE CITRATO, ALDACTAZIDE®, PROPAFENONE, DELTACORTENE®, CLOROCHINA BAYER®, CLEXANE®, LIMPIDEX®, AUGMENTIN®, ENDOPROST®	Positive	NR	Possible
55	F	METOCAL®	270	260	Epigastralgia	No	Recovered/resolved	SETRILAN®	Positive	Positive	Probable/likely
78	F	METOCAL®	7	7	Urticaria	No	Recovering/resolving	NR	Positive	NR	Probable/likely
55	F	METOCAL®	7	7	Urticaria	No	Recovering/resolving	NR	Positive	NR	Probable/likely
49	F	NATECAL D3®	0	0	Bronchospasm, Rash	Yes	Recovered/resolved	EUTIROX®	Positive	NR	Possible
73	F	CALCIO CARBONATO VITAMINA D3 EG®	16	10	Skin Exfolian	No	Recovered/resolved	ALENDROS®, FOSAMAX®	Positive	NR	Possible
68	F	OSSEOR®, TACHIPIRINA®, CACIT®	8	7	Steven-Johnson Syndrome	Yes	Recovering/resolving	NR	Positive	NR	Possible
55	M	DIBASE®, CALCIUM SANDOZ®	9	0	Urticaria	No	Recovered/resolved	NR	Positive	NR	Possible
51	F	FOSAMAX®, IDEOS®	42	28	Skeletal Pain, Fever Chills	No	Recovered/resolved	NR	Positive	NR	Possible
67	F	IDEOS®, DRONAL®	6	7	Nausea	No	Recovered/resolved	NR	Positive	NR	Possible

66	F	DIBASE ®, LANSOPRAZOLO HEXAL®, LOSAPREX®	46	46	Limb Rash	No	Recovering/resolving	AROMASIN®, TOLEP®, TIKLID®, TRITTICO®	Positive	NR	Possible
80	F	CALCITRIOLO EG®	2	2	Gastritis, Stomach Acid	No	Recovered/resolved	AMIODAR®, ANTACAL®, AULIN®, CARDIOASPIRIN®, CALCIUM SANDOZ®, SALMETEDUR®	Positive	NR	Possible
11	F	CALCIO CARBONATO VITAMINA D3 EG®, ROCALTROL®	3	3	Gastralgia, Rash, Vomiting	No	Recovered/resolved	EUTIROX®	Positive	NR	Possible
69	F	OSSEOR®, DISEON®, CRESTOR®	NR	15	Pruritus, Rash	No	Recovered with sequelae	NR	NR	NR	Possible
57	F	IDEOS®	1	1	Gastralgia, Vomiting	No	NR	OPTINATE®, LIVIAL®	NR	NR	Possible
82	M	CALCIO CARBONATO + VITAMINA D3 GERMED®	327	308	Pruritus	No	Recovering/resolving	LANSOX®, TIKLID®, NAPRILENE®, GARDENALE®, RESPICUR®, SPIRIVA®, SYMBICORT®, TACHIDOL®, MEDROL®	Positive	Positive	Probable/likely
68	F	NATECAL D3®	18	18	Eye Inflammation	No	Recovering/resolving	NAPRILENE®, CARDIRENE®, TORVAST®	Positive	Positive	Possible
72	F	DIBASE®	335	303	Diarrhoea, Dizziness, Dry Heaves	No	Recovered/resolved	EUTIROX®, PANTORC®, LASIX®, DELTACORTENE®, PLAQUENIL®	Positive	Positive	Probable/likely
57	F	IDEOS®	245	245	Pigmentation	No	Recovered/resolved	NR	Positive	NR	Probable/likely

54	F	CALCIUM D3 SANDOZ®	46	0	Dyspepsia, Breathing Impairment	No	Recovered/resolved	FOSAMAX®	Positive	NR	Possible
63	F	ROCALTROL®	7	0	Angioedema, Hand Pruritus	No	NR	NR	NR	NR	Probable/likely
68	F	CACIT®, DIBASE®	1096	1019	Gastralgia	No	Recovered/resolved	EUTIROX®, CARDIOASPIRIN®, TOTALIP®	Positive	NR	Possible
72	F	IDEOS®	10	1	Erythema	No	Recovered/resolved	ALENDRONATO SODICO	Positive	Positive	Probable/likely
47	M	DIBASE ®, FORSTEO®, TESTO ENANT®	21	21	Cutaneous Rash	Yes	NR	NR	NR	NR	Possible
69	F	CALCIUM D3 SANDOZ®	960	960	Persistent Nonproductive Cough	No	Recovered/resolved	NAPRILENE®, DEDRALEN®, TORVAST®	Positive	NR	Possible
76	F	ROCALTROL®	199	9	Epigastralgia, Burning With Regurgitation	No	NR	FERRO GRAD C®, LANSOPRAZOLO	NR	Positive	Possible
66	F	DIBASE®	0	0	Diarrhoea, Headache	No	Recovered/resolved	CARDIOASPIRIN®, NORMIX®, CARVIPRESS®	Positive	Positive	Probable/likely
56	F	CACIT®	10	5	Glossitis, Ulcerated Mouth	No	Recovered/resolved	NR	Positive	Positive	Probable/likely
78	F	DIFIX®	731	731	Hypercalcaemia	Yes	Recovering/resolving	LASIX®, TIKLID®, ZYLORIC®, ZOTON®, TILDIEM®, CALCIO CARBONATO EG®, EUTIROX®	Positive	NR	Possible
73	F	DIBASE®	0	0	Headache, Hypercalcaemia, Localized Rash	No	Persistent	DIFOSFONAL®	Negative	NR	Probable/likely
64	F	DIBASE®	63	45	Right Hypochondrium Pain, Petechiae	No	Recovered/resolved	NR	Positive	NR	Possible

68	F	DEDIOL	5	5	Conjunctival And Eyelid Oedema	No	Recovering/resolving	NR	Positive	NR	Possible
68	F	DEDIOL	5	8	Facial Redness, Malaise	No	Recovering/resolving	NR	Positive	NR	Probable/likely
11	F	CALCITRIOLO TEVA®, RIFAXIMINA, CEFTRIAXONE, CLARITROMICINA	0	0	Diffuse Erythema	No	Recovering/resolving	NR	Positive	NR	Probable/likely
87	F	IDEOS®	0	0	Constipation	No	Recovering/resolving	NR	Positive	NR	Probable/likely
50	F	NATECAL D3®	200	30	Pruritus	No	Recovering/resolving	MEDROL®	Positive	Positive	Probable/likely
79	F	OROTRE®	8	2	Joint Pain	No	NR	SEREVENT®	NR	NR	Possible
78	F	CACIT®	7	0	Hypertensive Crisis	NR	Recovering/resolving	TRIATEC®, TICLOPIDINA	Positive	Positive	Possible
76	F	DIBASE®	0	1	Facial Erythema, Angioedema, Rash	No	Recovered/resolved	NEXIUM®, CARDIOASPIRIN®, CALCIUM SANDOZ, NORMASE®	Positive	NR	Possible
60	M	CACIT®	8	0	Hypertension	No	Recovered/resolved	NR	Positive	NR	Probable/likely
69	F	DIBASE®	21	49	Asthenia, Dizziness, Nausea	No	Recovered/resolved	VASEXTEN®, CRESTOR®	Positive	NR	Possible
70	F	CALCIO CARBONATO VITAMINA D3 EG®	222	0	Dyspepsia	No	NR	EUTIROX®, DIDROGYL®, ALENDRONATO DOC®	Positive	NR	Possible
87	F	ROCALTROL®	1	1	Diffuse Erythema	No	Recovering/resolving	NITRO-DUR®, AMIODARONE, PANTOPRAZOLO, METOCLOPRAMIDE, CALCIO GLUCONATO	Positive	NR	Possible
48	F	DIFIX®, CALCIO CARBONATO EG®	3	3	Urticaria	No	Recovered/resolved	NR	Positive	NR	Possible

82	F	CALCIUM SANDOZ®, ROCALTROL®	295	295	Abnormal Behaviour, Hypercalcaemia	Yes	Recovered/resolved	LASIX®, TRIATEC®, LANSOX®, ARANESP®	Positive	Positive	Probable/likely
49	F	CALCIO CARBONATO, ROCALTROL®	242	242	Hypercalcaemia (Chronic Overdose)	No	Recovering/resolving	ADALAT®, RANIDIL®, EPOETINA ALFA	Positive	NR	Possible
48	F	DIFIX®, ZADITEN®	NR	NR	Acute Hepatitis	Yes	Persistent	NR	Negative	NR	Possible
92	F	DIBASE®	24	0	Diarrhoea	No	Recovered/resolved	NR	Positive	Positive	Probable/likely
87	F	IDEOS®, CARDIOASPIRIN®, DILATREND®	12	11	Erythema, Pruritus	No	NR		NR	NR	Possible
79	F	NATECAL D3®	2	2	Oral Inflammation And Desquamation	No	NR		NR	NR	Possible
73	F	IDEOS®	3	0	Nocturna Polyuria	No	Recovered/resolved		Positive	NR	Probable/likely
89	M	CALCITRIOLO DOC GENERICI ®, COUMADIN	827	828	Increased INR	No	Recovered/resolved		NR	NR	Possible
49	F	NATECAL D3®, DIBASE®	5	4	Gastralgia, Mouth Burning, Facial Desquamation	No	Recovered/resolved		Positive	NR	Possible
4 Months	F	DIBASE®	119	119	Infantile Colic, Restlessness, Astringent Therapy	No	Recovered/resolved		Positive	NR	Probable/likely
76	F	METOCAL®	14	5	Constipation (Excl Faecaloma)	No	NR		NR	NR	Possible
76	F	DIBASE®	0	1	Urticaria	No	Recovering/resolving		Positive	NR	Possible
44	F	DIBASE®	47	2	Nausea, Vomiting	No	NR		NR	NR	Possible
81	F	NATECAL D3®	4	4	Dyspepsia,Epigastric Burning	No	NR		NR	NR	Probable/likely

83	F	DIBASE®	468	468	Hypercalcaemia	Yes	NR		NR	NR	Probable/likely
51	F	NATECAL D3®	124	0	Gastralgia, Nausea	No	NR		NR	NR	Possible
1 Months	F	ADISTEROLO	10	10	Constipation, Irritability (Overdose)	Yes	Recovering/resolving		Positive	NR	Probable/likely
87	M	CALCIO CARBONATO EG®, CALCITRIOLO	564	564	Hypercalcaemia	Yes	Recovering/resolving		Positive	NR	Possible
52	F	CALCIO CARBONATO + VITAMINA D3 RATIOPHARM®	16	0	Stomach Cramps, Gastroesophageal Burning	No	NR		NR	NR	Possible
64	F	DIBASE®	30	0	Hyperidrosis, Nausea, Vomiting	No	NR		NR	NR	Probable/likely
59	F	DIBASE®	17	16	Dermatitis	No	Recovering/resolving		Positive	NR	Probable/likely
63	F	CALCIO CARBONATO VITAMINA D3 EG®	163	31	Gastrointestinal Alteration, Aerophagy	No	Persistent		NR	NR	Possible
62	F	CACIT®	1881	1614	Astringent Therapy	No	NR		NR	NR	Possible
56	F	CACIT®	4	4	Diarrhoea	No	Recovered/resolved		Positive	NR	Probable/likely
51	F	DIBASE®	29	29	Abdominal Pain, Swollen Abdomen	No	Recovering/resolving		Positive	NR	Possible
64	F	CALCICOLD3®	61	61	Abdominal Burning, Abdominal Pain, Vomiting	No	Recovered/resolved		Positive	NR	Possible
61	F	NATECAL D3®	28	22	Localised Urticaria	No	Recovered/resolved		Positive	NR	Probable/likely
77	F	DIBASE®	3	3	Gastric Pain, Hypertension	No	Recovered/resolved		Positive	NR	Possible
88	F	DIBASE®	0	0	Acute Confusional State	No	Recovered/resolved		Positive	NR	Probable/likely

64	F	DIBASE®	0	0	Renal Colic	No	NR		NR	NR	Possible
70	F	DIBASE®	0	0	Abdominal Pain, Diarrhoea	No	Recovered/resolved		Positive	NR	Possible
67	F	CALCIO CARBONATO VITAMINA D3 EG®	0	0	Eczema	No	Persistent		Negative	NR	Possible
65	F	METOCAL®	0	0	Allergic Urticaria	No	Recovering/resolving		Positive	NR	Possible
86	F	IDEOS®	2	2	Balance Loss	No	NR		NR	NR	Possible
67	F	METOCAL®	1240	1240	Nausea	No	Recovering/resolving		Positive	NR	Probable/likely
79	F	DIBASE®	0	0	Headache	No	NR		NR	NR	Probable/likely
63	F	CALCIO CARBONATO + VITAMINA D3 GERMED®	1	1	Abdominal Pain, Diarrhoea	No	Recovered/resolved		Positive	NR	Possible
58	F	CALCIO CARBONATO + VITAMINA D3 ABC FARMACEUTICI®	2	2	Diarrhoea, Nausea	No	NR		NR	NR	Possible
80	F	CALCIO CARBONATO + VITAMINA D3 RATIOPHARM®	1	1	Gastric Pain	No	NR		NR	NR	Possible
70	F	CACIT®	14	0	Hypertension, Dizziness	No	Recovered/resolved		Positive	NR	Possible
61	F	CACIT®	15	15	Dyspepsia	No	Recovered/resolved		Positive	NR	Probable/likely
78	F	CALCIO CARBONATO + VITAMINA D3 ABC FARMACEUTICI®	76	0	Vomiting	No	Recovered/resolved		NR	NR	Probable/likely
80	M	CALCIO CARBONATO + VITAMINA D3 ABC FARMACEUTICI®	3	3	Gastric Pain, Nausea	No	Recovered/resolved		Positive	NR	Possible
85	F	TONACAL D3®, DEPALGOS®	10	7	Erythema, Tremor	No	Recovered/resolved		Positive	NR	Possible
5 Months	F	DIBASE®	0	0	Eyelid Hyperemia, Rash	No	NR		NR	NR	Probable/likely
70	F	DIBASE ®, ROCALTROL®	5	5	Headache Worsening	No	NR		NR	NR	Probable/likely
72	F	DIBASE®	31	31	Cough, Dyspnea	No	Recovering/resolving		Positive	Positive	Probable/likely

71	M	CACIT®	6	6	Abdominal Pain, Constipation And Diarrhoea Alternation	No	Recovered/resolved		Positive	NR	Probable/likely
70	F	DIBASE®	6	6	Asthenia, Confusional State, Pruritus	No	Recovering/resolving		Positive	Positive	Probable/likely
45	M	NATECAL D3®	0	0	Pruritus, Maculopapular Rash	No	Recovering/resolving		Positive	NR	Probable/likely
60	F	CACIT ®, OSSEOR®	15	15	Retrosternal Pain, Pruritus, Rash	Yes	Recovering/resolving		Positive	NR	Possible
85	F	DIBASE®, COUMADIN®	2467	2454	Increased INR	No	Recovered/resolved		NR	NR	Possible
76	M	DIBASE®, CALCITRIOLO	730	730	Hypercalcaemia, Acute And Chronic Renal Failure	Yes	Recovered/resolved		Positive	NR	Possible
39	F	ROCALTROL®, CALCIO CARBONATO	72	72	Hypercalcaemia	No	Recovered/resolved		Positive	NR	Possible
81	F	NATECAL D3®	1	0	Diarrhoea, Dizziness, Nausea, Vomiting	No	Recovered/resolved		Positive	NR	Possible
67	M	DIBASE®	3	3	Epigastric Pain, Pyrosis (Drug Error Use)	No	Recovered/resolved		Positive	NR	Possible
2 Months	M	DIBASE®	0	0	Vomiting	No	NR		NR	NR	Probable/likely
81	F	DIBASE®	3	3	Joint Pain, Hyperpyrexia	No	Recovered/resolved		Positive	NR	Probable/likely
56	M	CALCIUM SANDOZ®, ROCALTROL®, ANTRA®	895	895	Hypercalcaemia	Yes	Recovered/resolved		Positive	NR	Possible
11 Months	F	ADISTEROLO	343	343	High Calcium (Drug Intoxication)	No	Recovering/resolving		Positive	NR	Probable/likely

65	F	DIBASE®	1	1	Gingival Disease	No	Recovering/resolving		Positive	NR	Probable/likely
73	F	CALCITRIOLO, CALCIO CARBONATO	44	44	Confusional State, Hypercalcaemia, Syncope	Yes	Recovered/resolved		Positive	Positive	Probable/likely
74	F	NATECAL D3®	9	2	Angioedema	No	Recovering/resolving		Positive	NR	Probable/likely
68	M	CALCITRIOLO TEVA®	85	85	Hyperglicaemia	No	Recovering/resolving		Positive	NR	Possible
74	F	METOCAL®	8	8	Urticaria	No	Recovered/resolved		Positive	NR	Possible
56	F	DIBASE®	0	0	Headache, Hypertension, Tachycardia, Hot Flash	No	Recovered/resolved		Positive	NR	Probable/likely
73	F	CALCITRIOLO, METOCAL	215	215	Coma, Epileptic Crisis, Hypercalcaemia	Yes	Recovered with sequelae		Negative	NR	Possible
43	F	METOCAL®	603	529	Brurning With Reflux	No	Recovered/resolved		Positive	Positive	Possible
73	M	CACIT®	63	63	Aggression, Confusion, Disorientation, Hypercalcaemia	Yes	Recovering/resolving		Positive	NR	Possible
94	F	KARVEA®, ROCALTROL®	60	60	Hypercalcaemia, Worsened Renal Failure	Yes	Recovering/resolving		Positive	NR	Possible
52	F	DIBASE®	0	0	Hypoaesthesia Oral	No	Recovered/resolved		Positive	NR	Probable/likely
76	M	ATITEN®	0	0	Dehydration, Hypercalcaemia, Muscular Spams, Vomiting Heaves, Drowsiness, Acute Renal Failure	Yes	Recovered/resolved		Positive	NR	Probable/likely

37	M	DIBASE®	34	34	Hypercalcaemia	Yes	Recovering/resolving		Positive	NR	Possible
51	F	CACIT®	3	3	Oedema	No	Recovering/resolving		Positive	NR	Probable/likely
70	F	DIBASE®, ROCALTROL®	0	0	Hypertensive Encephalopathy	Yes	Recovered/resolved		Positive	NR	Possible
54	F	CACIT®	2644	2621	Diarrhoea	No	NR		NR	NR	Possible
77	F	DIFIX®, CONTRAMAL®, TACHIDOL®, NATECAL D3®	5	2	Abnormal Creatinine, Worsening, Allergic Reaction	Yes	Recovering/resolving		Positive	NR	Possible
60	F	DIBASE®, NERIXIA®	0	2	Joint Pain, Injection Site Inflammation	No	Recovering/resolving		Positive	NR	Possible
78	F	CACIT ®, ROCALTROL®, NATECAL D3®	52	52	Hypercalcaemia, Reduced General Conditions, Hyporexia (Drug Intoxication)	Yes	Recovered/resolved		Positive	NR	Possible
41	F	CALCIUM SANDOZ®, ROCALTROL®	85	74	Urticaria	No	Persistent		Negative	NR	Possible
65	F	METOCAL®	7	7	Abdominal Pain, Dyspepsia	No	Recovered/resolved		Positive	Positive	Probable/likely
84	F	CACIT ®, MIRTAZAPINA MYLAN GENERICS®, LANOXIN®	422	422	Vomiting, Nausea	No	Recovered/resolved		Positive	NR	Possible
70	F	NATECAL D3®, PLAQUENIL®	27	24	Diffuse Erythema And Pruritus	No	Recovering/resolving		Positive	NR	Possible
64	F	DIBASE®	53	84	Hypercalcaemia	Yes	Recovered/resolved		NR	NR	Possible
73	F	KALAZD3®	260	260	Diarrhoea	No	Recovered/resolved		Positive	NR	Possible
55	F	IDEOS®, BRUFEN®, GASTROLOC®	0	10	Dermatosis	Yes	Recovering/resolving		Positive	NR	Possible

86	F	IDEOS®	91	91	Hypercalcaemia	No	Recovering/resolving		NR	NR	Possible
48	F	NATECAL D3®	20	20	Depression, Nervousness	No	Recovered/resolved		Positive	NR	Possible
59	F	CACIT®	750	906	Esophageal Candidiasis	No	Recovering/resolving		NR	NR	Possible
59	F	NATECAL D3®	669	700	Gingival Bleeding, Toothache	No	Recovered/resolved		Positive	Positive	Probable/likely
53	F	CALCIO CARBONATO/COLECALCIFEROLO	10	10	Pruritus, Urticaria	No	Recovering/resolving		Positive	NR	Probable/likely
59	F	DIBASE®	0	1	Rash	No	Recovering/resolving		Positive	NR	Probable/likely
62	F	ROCALTROL®, CALCIUM SANDOZ®	617	617	Hypercalcaemia	Yes	Recovered/resolved		Positive	NR	Possible
51	F	DIBASE®	0	4	Asthenia, Confusional State, Dry Mouth, Euphoric Mood, Fatigue, Hyperreflexia	Yes	Recovered/resolved		NR	NR	Possible
74	F	DIBASE®	3	4	Chest Constriction, Dry Mouth, Mood Disorder, Nausea, Somnolence, Systolic Hypertension	No	Recovering/resolving		NR	NR	Possible
54	F	DIBASE®	NR	1	Acute Abdominal Pain	No	Persistent		NR	NR	Probable/likely
68	F	DIBASE®, OSTEUM®	3	0	Nausea, Vomiting, Vertigo	Yes	Recovering/resolving		Positive	NR	Possible
53	F	DIBASE®	1	1	Diarrhoea, Nausea, Vertigo	No	Recovered/resolved		Positive	NR	Possible
58	F	DIBASE®	14	16	Urticaria	No	Recovered/resolved		NR	NR	Possible

51	F	DIBASE ®, CACIT ®, BRUFEN®	916	899	Decreased Hemoglobin	Yes	Persistent		Negative	NR	Possible
74	F	DIBASE®	0	0	Angioedema, Throat Tightness	No	Recovering/resolving		Positive	NR	Possible
66	F	DIBASE®, BONVIVA®	1218	1117	Urticaria	No	Recovering/resolving		Positive	NR	Possible
55	F	DERIL®	39	39	Agitation, Tachycardia	No	Recovered/resolved		Positive	NR	Probable/likely
78	M	ROCALTROL®	0	0	Rash	No	Recovering/resolving		Positive	NR	Possible
76	M	DIBASE ®, COUMADIN®, PARACODINA®, DOBETIN®, FOLINA®	2304	2297	Decreased INR	No	Recovered/resolved		Positive	NR	Possible
83	F	DIBASE®, OROTRE®	0	0	Agitation, Chest Pain, Palpitations	No	Recovered/resolved		Positive	Positive	Possible
87	F	CALCITRIOLO TEVA ®, NATECAL D3®	16	16	Hypercalcaemia	Yes	NR		NR	NR	Possible
74	F	NATECAL D3®, CALCIO CARBONATO/COLECALCIFEROLO,	280	0	Abdominal Swelling, Constipation	No	Persistent		NR	NR	Possible
58	M	DIBASE®	0	0	Feeling Abnormal	No	Recovered/resolved		Positive	NR	Possible
85	F	OSTELIN®	526	532	Hypercalcaemia (Chronic Overdose)	Yes	Recovering/resolving		Positive	NR	Probable/likely
67	F	CALCIUM D3 SANDOZ®	0	0	Diarrhoea	No	Recovered/resolved		Positive	NR	Probable/likely
74	F	CALCIUM SANDOZ®, ROCALTROL®	3	3	Hypercalcaemia	Yes	NR		NR	NR	NR
84	F	METOCAL®	321	60	Dyspepsia	No	Persistent		NR	NR	NR
24 Days	M	DIBASE®	0	0	Increased Urinary Calcium/Creatinine Ratio, Regurgitation	No	Recovered/resolved		Positive	NR	Probable/likely
84	F	DIBASE®, OSSEOR®	1	1	Facial Erythema And Warmth, Angioedema	No	Recovering/resolving		Positive	NR	Possible

85	F	CALCIO CARBONATO E VITAMINA D3 SOFAR®	0	0	Unusual Drug Solubility	No	NR		NR	NR	Unlikely
48	M	DIBASE®	132	41	Nausea, Vomiting	No	NR		NR	NR	Possible
74	F	DIBASE®	7	0	Somnolence	No	Recovered/resolved		Positive	NR	Possible
79	F	CACIT®	10	10	Dyspepsia	No	Recovered/resolved		Positive	NR	Possible
60	F	DIBASE®	0	0	Cutaneous Rash	No	Recovering/resolving		Positive	NR	Probable/likely
95	F	DIBASE ®, FORSTEO®, RISEDRONATO SODICO	35	39	Increased Gamma-GT And Transaminasis, Hypercalcaemia, Pruritus	No	NR		NR	NR	Possible
77	F	DIFIX®	183	183	Hypercalcaemia	No	Recovering/resolving		Positive	NR	Possible
78	F	FOSCALD3®	11	4	Abdominal Pain, Appetite Loss	No	Recovering/resolving		Positive	NR	Possible
77	F	ROCALTROL®, CACIT®	37	37	Hypercalcaemia (Therapeutic Error)	Yes	Recovered/resolved		Positive	NR	Possible
74	F	CALCIO CARBONATO VITAMINA D3 EG®	158	158	Nausea, Vomiting	No	Recovered/resolved		Positive	Positive	Probable/likely
55	F	CALCITRIOLO	247	244	Tachyarrhythmia	No	Recovering/resolving		Positive	NR	Probable/likely
48	M	DIBASE®, ATRIPLA®	437	437	Apathy, Asthenia, Depression, General Malaise, Appetite Loss	No	Recovered/resolved		NR	NR	Possible
42	F	DIFIX®	42	26	Constipation	No	Recovered/resolved		Positive	Positive	Probable/likely
76	M	CALCITRIOLO, COUMADIN®, XELEVIA®	268	269	Increased INR	No	Recovered/resolved		Positive	NR	Possible
72	F	DIBASE®, NATECAL D3®	72	72	Confusion, Hypercalcaemia, Drowsiness	Yes	Recovered/resolved		NR	NR	Possible

72	F	DIBASE. NATECAL D3®	72	72	Confusion, Hypercalcaemia, Drowsiness	Yes	Recovered/resolved		Positive	NR	Possible
44	F	CALCIUM SANDOZ®, ROCALTROL®	NR	NR	Hypercalcaemia	Yes	Recovering/resolving		Positive	Positive	Probable/likely
74	F	NATECAL D3®, BONASOL®, PRITORPLUS®, PARACETAMOLO	663	639	Urticaria	No	Recovering/resolving		Positive	NR	Possible
54	F	DIBASE®	0	0	Diarrhoea	No	Recovered/resolved		Positive	NR	Possible
57	M	DIBASE®	NR	8	Hypertension, Insomnia	No	Persistent		Negative	NR	Possible
63	F	DIBASE®	132	132	Amnesia, Confusion, Horthostatic Hypotension	No	Recovered/resolved		Positive	NR	Possible
59	M	CACIT®	21	21	Abdominal Swelling	No	Recovered/resolved		Positive	NR	Possible
81	F	COLECALCIFEROLO, PREDNISONE	364	364	Confusional State, Presyncope	Yes	Persistent		NR	NR	Possible
71	F	DIBASE®	62	0	Headache	No	Recovering/resolving		Positive	NR	Possible
60	F	NATECAL D3®	5	0	Diarrhoea	No	Recovered/resolved		Positive	NR	Probable/likely
52	M	ROCALTROL®, AMIODAR®	18	15	Pruritus	No	Recovered/resolved		Positive	NR	Possible
73	F	DIBASE®	0	1	Angioedema	No	Recovered/resolved		Positive	NR	Possible
81	F	NATECAL D3®, BENTELAN®, CETIRIZINA DICLORIDRATO	37	37	Diffuse Erythema And Pruritus	No	Recovered/resolved		Positive	NR	Possible
39	F	DEDIOL®, CARDIOASPIRIN®, EXINEF®, LANSOPRAZOLO	926	926	Duodenal Ulcer, Melaena, Presyncope	Yes	Recovering/resolving		Positive	NR	Possible
23	M	MESAVANCOL®, CALISVIT®	240	241	Rash	No	Recovering/resolving		Positive	NR	Possible

NR	F	DIBASE®	NR	NR	Diarrhoea	NR	NR		NR	NR	Possible
4 Months	M	DIBASE®	7	0	Vomiting, Regurgitation (Therapeutic Error)	Yes	Recovered/resolved		Positive	NR	Probable/likely
81	F	CALCITRIOLO EG®, COUMADIN®, TACHIDOL®	895	895	Increased INR	No	Recovered/resolved		Positive	NR	Possible
74	F	NATECAL D3®, DIBASE®	14	13	Urticaria	No	Recovered/resolved		Positive	NR	Possible
65	F	CACIT®	NR	NR	Subcutaneous Cyst, Alterate Skin Sensitivity	NR	NR		NR	NR	Possible
49	F	DIBASE®	93	1	Acute Diarrhoea	No	Persistent		Positive	NR	Possible
67	F	DIBASE®	26	6	Diffuse Erythema And Pruritus	No	Recovering/resolving		Positive	NR	Possible
24	F	DIBASE®	11	11	Rash	No	Recovering/resolving		Positive	NR	Probable/likely
64	F	ROCALTROL®, CALCIO CARBONATO	112	112	Headache, Hypercalcaemia, Hypokalaemia, Nausea, Acute Renal Failure (Drug Intoxication)	Yes	Recovered/resolved		Positive	Positive	Probable/likely
64	F	DERIL®	71	71	Abdominal Swelling And Pain, Flatulence	No	Recovering/resolving		Positive	NR	Possible
79	F	DIBASE®	0	0	Cutaneous Rash	No	Recovering/resolving		Positive	NR	Possible
59	F	DIBASE®, FEMARADILATREND®, LOSAPREX®, XANAX®, DELTACORTENE®, PARACODINA®	0	0	Drowsiness (Drug Abuse)	Yes	Recovered/resolved		NR	NR	Possible
56	F	DIBASE®	0	0	Diarrhoea	No	Recovered/resolved		Positive	NR	Probable/likely

58	F	NATECAL D3®	0	0	Asthenia, Throat Tightness	No	Recovering/resolving		Positive	NR	Probable/likely
75	F	DIBASE®	36	6	Dermatitis, Pruritus	No	Recovered/resolved		Positive	NR	Possible
7 Months	M	DIBASE®	NR	NR	Unwanted Drug Ingestion	Yes	NR		NR	NR	Unlikely
81	F	DIBASE®	0	5	Limb Burning	No	Recovering/resolving		Positive	NR	Possible
70	F	ROCALTROL®, CALCIUM SANDOZ®	2	2	Hypercalcaemia	Yes	Recovered/resolved		Positive	NR	Possible
NR	F	DIBASE®	50	NR	Hot Flashes	No	Recovered/resolved		Positive	Negative	Possible
46	M	CALCIO CARBONATO, ROCALTROL®	102	102	Hypercalcaemia	Yes	Recovered/resolved		Positive	NR	Possible
75	F	NATECAL D3®, ARAVA®, COUMADIN®	375	375	INR Increased	No	Recovered/resolved		NR	NR	Possible
20	M	DIBASE®	6	6	Nausea, Spasms (Drug Abuse)	No	Recovering/resolving		Positive	NR	Probable/likely
24	F	DIBASE®	10	5	Abdominal Cramps, Constipation, Urination Difficulty, Increased Thirst	No	Recovered/resolved		Positive	NR	Probable/likely
62	F	DIBASE®	1	1	Headache, Nausea, Vomiting	No	Recovered/resolved		Positive	NR	Probable/likely
78	F	DIBASE®	NR	0	Dorsal Pain	No	Recovering/resolving		Positive	Positive	Probable/likely
66	F	DIBASE®	NR	7	Constipation, Hemorrhoids	No	NR		NR	Positive	Probable/likely
55	F	DIBASE®	NR	NR	Jaundice	No	NR		NR	Negative	Possible
68	F	DIBASE®	0	0	Dyspnea, Formication, General Malaise, Presyncope, Vertigo	No	Recovered/resolved		Positive	NR	Possible

62	F	NATECAL D3®, ACTONEL®, DIBASE®	1848	1848	Spine Fracture (Pharmacokinetic Interaction)	Yes	Recovering/resolving		NR	NR	Possible
64	F	CACIT ®, COUMADIN®	4271	4271	Increased INR (Pharmacokinetic Interaction)	No	Recovered/resolved		NR	NR	Possible
2 Months	F	DIBASE®	31	31	Tachycardia (Drug Administration Error)	No	Recovering/resolving		Positive	NR	Probable/likely
50	F	DIBASE®	761	365	Breast Tenderness	No	NR		NR	Positive	Probable/likely
67	F	DERIL®	7	7	Hypertension	No	Recovered/resolved		Positive	NR	Probable/likely
65	M	DIBASE®	28	28	Dermatitis, Erythema, Pruritus	No	Recovered/resolved		Positive	NR	Probable/likely
70	F	DIBASE®, CO EFFERALGAN®	0	0	Presyncope	No	Recovering/resolving		NR	NR	Possible
2	F	CALISVIT®	NR	NR	Constipation	No	Persistent		NR	NR	Possible
60	F	DELTIUS®	923	923	Xerostomia	No	Persistent		Positive	NR	Possible
79	F	DIBASE®, COUMADIN®	677	677	Increased INR	No	NR		NR	NR	Possible
32	F	DELTIUS®	0	0	Allergic Reaction, Pruritus, Rash	No	Recovering/resolving		Positive	NR	Possible
39	F	COLECALCIFEROLO	0	0	Diffuse Erythema	No	Recovering/resolving		Positive	NR	Possible
73	F	COUMADIN®, ADROVANCE®	1518	1518	Increased INR	No	NR		NR	NR	NR
70	F	DELTIUS®	0	1	Joint Pain, Finger Swelling	No	NR		NR	NR	Possible
88	F	CALCIO CARBONATO VITAMINA D3 EG®	5	3	Epigastralgia	No	Recovered/resolved		Positive	NR	Possible
85	F	DIBASE®, COUMADIN®, DELTACORTENE®, PLAQUENIL®	1919	1919	Increased INR (Pharmacokinetic Interaction)	Yes	Recovered/resolved		NR	NR	NR
59	F	OSTEOFOSD3®	61	61	Polyuria	No	Recovering/resolving		Positive	NR	Possible

2	F	DIBASE®	0	0	Abdominal Pain (Unwanted Use)	No	Recovering/resolving		NR	NR	Probable/likely
75	F	DIBASE®, CARDIOASPIRIN®	24	0	Epistaxis	No	Recovered/resolved		Positive	Positive	Possible
63	F	TREDIMIN®	76	19	Bitter Taste, Flank Pain, Nausea	Yes	NR		NR	Positive	Probable/likely
72	F	CALCIO FOSFATO TRIBASICO/COLECALCIFEROLO, DELTACORTENE, AZATIOPRINA	226	225	Epigastralgia	No	Recovering/resolving		NR	NR	NR
57	F	DIBASE ®, GILENYA®	187	182	Lung Adenocarcinoma	Yes	NR		NR	NR	Possible
NR	F	DIBASE®	NR	NR	Eczema Reaction, Gastrointestinal Disease, Nasal Pruritus	No	NR		NR	NR	Possible
46	F	DIBASE®, CACIT®	0	1	Abdominal Cramps, Headache Worsening, General Muscular Pain	No	Recovered/resolved		Positive	NR	Possible
71	F	DIBASE®, ANTABREST®	126	58	Facial Erythema And Pruritus, Angioedema, Allergic Reaction	No	Recovered/resolved		Positive	NR	Possible
79	F	DELTIUS®	0	0	Epigastralgia, Diarrhoea	No	Recovered/resolved		Positive	NR	Possible
81	F	DIBASE®, ACIDO IBANDRONICO	5799	5691	Jaw Osteonecrosis	Yes	Recovered with sequelae		NR	NR	Possible
NR	M	HARVONI®, KALETRA®, BACTRIM®, FOLINA®, DELTIUS®, LAMIVUDINA	NR	NR	Amnesia, Epileptic Crisis	Yes	NR		NR	NR	Possible
1	F	DIBASE®	31	31	Erythema	No	Recovering/resolving		Positive	NR	Probable/likely

5 Months	F	DIBASE®	NR	NR	Cutaneous Calcification	No	Persistent		NR	NR	Possible
27	F	DIBASE®	0	0	Rash, Allergic Pruritus	No	Recovering/resolving		Positive	NR	Probable/likely
56	M	DIBASE®, DELTIUS®	NR	NR	Rash	No	NR		NR	NR	Possible
NR	F	DIBASE®	NR	NR	Skeletal Pain	No	NR		NR	NR	Possible
81	M	DIBASE®	25	24	Dermatitis, Facial Oedema	No	Recovering/resolving		Positive	NR	Possible
3	F	DIBASE®	52	1	Crying, Insomnia, Irritability, Decreased Appetite	No	Recovered/resolved		Positive	NR	Possible
60	F	CACIT ®, EUTIROX®	NR	NR	Atrophic Vulvovaginitis, Vaginal Dryness	No	NR		Positive	NR	Probable/likely
93	F	OROTRE®	NR	NR	Urticaria	No	Persistent		NR	NR	Possible
65	F	DELTIUS®, FORSTEO®	NR	NR	Gastric Pain, Skeletal Pain, Colitis, Gastritis, Headache, Head Discomfort, Vertigo	NR	NR		NR	NR	Possible
90	F	DIBASE®	NR	1	Asthenia, Polyuria (Drug Abuse)	No	Recovered/resolved		NR	NR	Probable/likely
46	M	DERIL®	2	2	Hypertensive Crisis	No	Recovered/resolved		Positive	NR	Probable/likely
72	F	COLECALCIFEROLO, ASCRIPTIN®, DUROGESIC®, LAROXYL®, VALIUM®, PANTOPRAZOLO, AMLODIPINA, TIOTROPIO BROMURO, SALMETEROLO XINAFOATO/FLUTICASONE PROPIONATO	NR	NR	Mental Confusion, Disorientation	Yes	Recovering/resolving		NR	NR	Possible

79	F	DELTIUS®, LEPONEX®, NIFEREX®	84	56	Infection, Petechiae, Pruritus, Skin Wound	No	Recovered/resolved		Positive	NR	Possible
83	F	XARENEL®	4	0	Asthenia	Yes	NR		NR	NR	Probable/likely
35	F	DIBASE®	NR	NR	Eyelid Cutaneous Rash	No	Persistent		NR	NR	Possible
30	M	DIBASE®	NR	NR	Abdominal Swelling, Constipation, Headache, Fatigue, Dermatitis, Somnolence	No	NR		NR	NR	Probable/likely
NR	F	DIBASE®, SEROQUEL®, RISPERDAL®	NR	NR	Inefficacy, Dysuria, Allucinations, Insomnia, Paranoia (Pharmacokinetic Interaction)	No	NR		NR	NR	Possible
67	F	CACIT®	NR	NR	Myalgia	No	NR		Negative	NR	Probable/likely
69	F	COLECALCIFEROLO, CALCIO CARBONATO, CALCITRIOLO	NR	NR	Hypercalcaemia, Acute Renal Failure	Yes	Recovering/resolving		Positive	Positive	Probable/likely
41	F	IDEOS®, ZIRTEC®, EUTIROX®	0	0	Agitation (Therapeutic Error)	No	Recovering/resolving		Positive	NR	Possible
NR	F	DERIL®, VASEXTEN®, NEXIUM®, LASIX®, PLAUNAC®, RASILEZ®, COUMADIN®, ZOCOR®, LANOXIN®, MEDROL®	NR	NR	Blood Pressure Disorder	No	NR		NR	NR	Possible
71	M	ZIBENAK®	NR	0	Somnolence	No	Persistent		NR	NR	Possible
90	F	NATECAL D3®	NR	NR	Tongue Rash	No	Persistent		Negative	NR	Possible
86	F	DIBASE®, COUMADIN®, TACHIPIRINA®	6991	6973	Hematoma	No	NR		NR	NR	Possible

NR	M	DIBASE®	NR	NR	Tachycardia	No	NR		NR	NR	Possible
54	F	DERIL®	7	7	Low Abdominal Cramps, Headache	No	Recovered/resolved		Positive	NR	Probable/likely
83	F	CLODY®, FOSAVANCE®	3504	3504	Jaw Osteonecrosis	No	NR		NR	NR	Possible
56	F	DIBASE®	0	0	Diarrhoea	No	Recovered/resolved		NR	NR	Probable/likely
64	M	CALCITRIOLO	NR	NR	Urticarial Erythema	No	Recovered/resolved		Positive	NR	Probable/likely
65	F	XARENEL®	NR	NR	Diarrhoea	No	Recovered/resolved		NR	NR	Probable/likely
71	M	DIBASE®	30	30	Abnormal Electroencephalogram	No	NR		NR	NR	Possible
NR	M	CALCITRIOLO TEVA®	NR	NR	Dyspnea, Insomnia, Nausea (Intentional Overdose)	No	Recovered/resolved		NR	NR	Possible
57	F	DIBASE®, ZANEDIP®	951	920	Blood Pressure Increased, Headache, Tachycardia, Vertigo	No	Recovering/resolving		Positive	NR	Possible
60	F	DIBASE®, LAROXYL®, EUTIROX®, LIMPIDEX®	NR	NR	Vesical Tenesmus	NR	NR		NR	NR	Possible
72	F	CALCITRIOLO, CALCIO CARBONATO/COLECALCIFEROLO	290	291	Confusion, Hypercalcaemia, Presyncope	Yes	Recovered/resolved		Positive	NR	Possible
68	F	OPTINATE®, FOSAVANCE®	730	1826	Infection	Yes	Recovered/resolved		Positive	NR	Possible
85	F	NATECAL D3®	0	0	Sited Pain And Oedema	No	Recovered/resolved		Positive	NR	Possible
4 Months	F	CALISVIT®	NR	NR	Urticaria	No	NR		NR	NR	Possible

80	F	DIBASE®, LANSOX®, PRITOR®, SIMESTAT®, ACTONEL®, CARTEOL®, LETROZOLO	NR	NR	Asthenia, Hypertension, Malaise	Yes	NR		NR	NR	Possible
77	F	DIBASE®	0	0	Presyncope	Yes	Recovering/resolving		Positive	NR	Probable/likely
40	M	DIBASE®	NR	NR	Angioedema	Yes	Recovered/resolved		Positive	NR	Probable/likely
59	F	DIBASE®, COUMADIN®, FOLINA®, DOBETIN®	161	161	Increased INR	No	Recovered/resolved		NR	NR	Possible
NR	F	DIBASE®	NR	NR	Pruritus, Rash	Yes	Persistent		NR	NR	Possible
60	F	DIBASE®	NR	NR	Diarrhoea	No	Recovered/resolved		Positive	NR	Probable/likely
3	M	DIBASE®	NR	NR	Wheals (Unwanted Drug Ingestion)	Yes	Recovered/resolved		NR	NR	Probable/likely
88	F	DIBASE®, DIDROGYL®	92	92	Erythema, Pruritus	No	Recovering/resolving		Positive	NR	Possible
NR	M	ROCALTROL®	NR	NR	Headache, Midriasis, Tongue Swelling, Vision Blurred, Periocular Swelling	NR	NR		NR	NR	Possible
NR	F	CALCIO CARBONATO, ROCALTROL®	NR	NR	Allergic Reaction, Hypocalcaemia	Yes	NR		NR	NR	Possible
73	F	DELTACORTENE®, FOSAVANCE®, DIBASE®	1121	268	Jaw Disease, Dental Implant Failure	No	Persistent		NR	NR	Possible
2 Months	F	DIBASE®	NR	31	Hypercalcaemia, Hyporexia	Yes	Persistent		Negative	NR	Possible
66	F	BONVIVA®, FOSAVANCE®	365	1716	Jaw Osteonecrosis	Yes	NR		NR	NR	Possible
63	F	TOPAMAX®, FOSAVANCE®	1820	1758	Dysgeusia, Oropharingeal Burning	No	Recovering/resolving		Positive	NR	Possible

75	F	DIBASE®	NR	NR	Abdominal Pain, Vaginal Discharge	No	Recovered/resolved		Positive	NR	Probable/likely
53	F	DIBASE®, MOTILIUM®, LENTOKALIUM®, ANSIOLIN®, IBUPROFENE	0	0	Drowsiness, Intentional Self Harm (Drug Abuse)	Yes	Recovered/resolved		NR	NR	Possible
64	F	DIBASE®, DEPAKIN®, CITALOPRAM	NR	NR	Feeling Hot, Increased Lipase	No	Persistent		NR	NR	Possible
78	F	DIBASE®, BONASOL®, CONDRAL®, EUTIROX®, PROVISACOR®, SEQUACOR®	NR	NR	Cough, Skin Redness, Black Tongue, Pharinxlaringeal Disturb	No	Recovering/resolving		NR	NR	Possible
NR	M	ROCALTROL®	NR	NR	Pruritus	No	NR		NR	NR	Probable/likely
NR	F	DIBASE®, MEDEOROS®	NR	NR	Increased PTH	No	NR		NR	NR	Possible
57	M	COLECALCIFEROLO EG®, XARENEL®	NR	NR	Respiratory Fatigue	No	Persistent		NR	NR	Possible
29	M	CALCITRIOLO TEVA®	NR	NR	Pruritus, Rash	No	Persistent		NR	NR	Probable/likely
NR	F	CALCIO CARBONATO/COLECALCIFEROLO, FORSTEO®	NR	NR	Ankle Swelling, Weigh Increased	No	NR		NR	NR	Possible
67	F	XARENEL®	NR	NR	Fatigue, Dizziness	No	NR		NR	NR	Possible
66	F	FOSAVANCE ®, MEDROL®, DIBASE®,	51	51	General Malaise	No	Recovered/resolved		NR	NR	Possible
64	F	DIBASE®	3	1	Cutaneous Rash	No	Recovering/resolving		Positive	NR	Possible
NR	M	DIBASE®, CALCITRIOLO	NR	NR	Irritability, Muscular Pain And Stiffness	No	NR		NR	NR	Possible
NR	F	XARENEL®	NR	NR	Toothache	No	Recovered/resolved		Positive	NR	Possible
82	F	NATECAL D3®	NR	NR	Intentional Wrong Therapeutic Use	No	Recovered/resolved		NR	NR	Unlikely

89	F	DIBASE®	1	1	Urticaria	No	Recovering/resolving		Positive	NR	Possible
57	F	DIBASE®	0	0	General Malaise, Limb Tremor, Idiosyncratic Reaction	No	Recovering/resolving		NR	NR	Probable/likely
77	F	DIBASE®	NR	NR	Blood Pressure Increased	No	NR		NR	NR	Possible
78	F	DIBASE®	31	NR	Hyperidrosis (Overdose, Therapeutic Error)	No	Recovered/resolved		NR	NR	Possible
70	F	CACIT ®, GARDENALE®	26	23	Cutaneous Rash	No	Persistent		Positive	NR	Possible
51	F	TREDIMIN®	NR	1	Diarrhoea, Gastritis, Gastrointestinal Disease, Fever, Decreased Appetite	No	Persistent		NR	NR	Probable/likely
5	F	DIBASE®	1	1	Pruritus, Rash, Vomiting	No	Recovering/resolving		Positive	NR	Probable/likely
60	F	ZIBENAK®	NR	NR	Abdominal Pain, Diarrhoea	No	NR		NR	NR	Probable/likely
10	F	DELTIUS®	0	0	Facial Erythema And Warmth, Rhinitis	No	Recovered/resolved		Positive	NR	Probable/likely
72	F	CADTRE®	2	0	Diarrhoea	No	Recovering/resolving		NR	NR	NR
56	F	DIBASE®	NR	NR	Metallic Taste, Headache	No	NR		NR	NR	Probable/likely
33	F	COLECALCIFEROLO, TOPAMAX®, XANAX®, DIBASE®	0	0	Suicide Attempt, Drowsiness	Yes	NR		NR	NR	Possible
43	F	CACIT®	NR	NR	Epigastric Pain, Dyspepsia	No	Recovered/resolved		NR	NR	Possible
65	M	DIBASE®	NR	NR	Blurred Vision	No	NR		NR	NR	Possible
NR	F	DIBASE®, FOLINA®	494	NR	Gastric Pain, Flatulence,	No	Recovering/resolving		NR	NR	Possible

					Mood Impairment, Pruritus, Tinnitus, Leukorrhea						
17 Days	F	DIBASE®	12	12	Crying, Irritability, Vomiting (Therapeutic Error, Off Label Use)	Yes	Recovered/resolved		NR	NR	Probable/likely
68	M	NATECAL D3®, EUTIROX®, SILDENAFIL SANDOZ®	NR	NR	Nipple Swelling	No	NR		NR	NR	Possible
48	F	DIBASE®	NR	NR	Anxiety, Mental Distress, Fatigue, Oppression	No	NR		NR	NR	Probable/likely
65	F	COLECALCIFEROLO EG®	150	137	Diarrhoea	No	Recovered/resolved		Positive	Positive	Probable/likely
11	M	DIBASE®	NR	NR	Confusional State, Fear	No	Recovered/resolved		Positive	NR	Probable/likely
60	F	DIBASE®	0	0	Urticaria	No	Recovering/resolving		Positive	NR	Probable/likely
2	F	DIBASE®	0	0	Unwanted Drug Ingestion	No	NR		NR	NR	Probable/likely
65	F	ANNISTER®	0	5	Headache And Discomfort, Nausea, Pruritus	Yes	Recovered/resolved		Positive	NR	Probable/likely
88	F	ROCALTROL®, TENORMIN®, TRINITRINA, ACIDO ACETILSALICILICO	NR	NR	Postural Loss, Confusional State, Allucinations, Headache, Malaise, Vision Impairment	No	NR		NR	NR	Possible
55	F	COLECALCIFEROLO, CIMZIA®, BENTELAN®	NR	NR	Weight Increased, Nocturnal Hyperidrosis	Yes	Persistent		NR	NR	Possible

59	F	DIBASE®	NR	NR	Polydipsia	No	Recovered/resolved		NR	NR	Probable/likely
NR	F	ROCALTROL®	NR	NR	Fatigue, Migraine, Tachycardia (Therapeutic Error)	No	NR		NR	NR	Probable/likely
NR	M	NATECAL D3®	NR	NR	Hypercalcaemia, Hypoalbuminemia	No	Recovered/resolved		Positive	NR	Probable/likely
NR	F	DIBASE®	NR	NR	Mouth Burning, Unusual Drug Taste	No	NR		NR	NR	Possible
NR	M	DIBASE®	NR	NR	Mouth Burning, Unusual Drug Taste	No	NR		NR	NR	Probable/likely
71	M	NATECAL D3®	3	0	Head Discomfort, Insomnia, Pruritus	No	Recovered with sequelae		Positive	NR	Probable/likely
80	F	DIBASE®	21	0	Diarrhoea, Xerostomia	No	Recovered/resolved		Positive	Positive	Probable/likely
69	F	BONVIVA®, FOSAVANCE®	3652	4717	Fistula, Gingival Suppuration, Jaw Osteonecrosis	Yes	Persistent		Negative	NR	Possible
45	F	DIBASE®	0	0	Asthenia, Somnolence	No	NR		NR	NR	Probable/likely
75	M	ROCALTROL®, SPIOLTO®, RESPIMAT®, CALCIO CARBONATO, AMLODIPINA SODIO BICARBONATO, LASIX®	NR	NR	Acute Respiratory Failure, Pneumonia, Chronic Renal Failure Worsening	Yes	Recovered/resolved		Positive	NR	Possible
74	F	ZIBENAK®	NR	NR	Pruritus	No	Persistent		Negative	NR	Probable/likely
NR	F	DIBASE®	NR	NR	Rash	No	Persistent		NR	NR	Probable/likely

53	F	DIBASE®	1651	1651	Urticaria	No	Recovered/resol ved		Positive	NR	Possible
67	F	LAMPARD®	3	0	Gingival Bleeding	No	Recovered/resol ved		Positive	Negative	Possible
65	M	ROCALTROL®, DIBASE®	7	7	Dermatitis, Rash	No	NR		Positive	NR	Possible
55	F	BRUFEN®, ANNISTER®	0	1	Pruritus, Angioedema, Acute Urticaria	Yes	Recovered/resol ved		Positive	NR	Possible
9	M	CACIT®	4	1	Maculo-Papular Exanthema	Yes	Recovering/res olving		Positive	NR	Probable/li kely
51	F	COLECALCIFEROLO	0	0	Pharyngolaryng eal Discomfort	No	Recovered/resol ved		Positive	NR	Probable/li kely
NR	F	DIBASE®	NR	NR	Throat Burning, Unusual Drug Taste	No	NR		NR	NR	Possible
NR	F	DIBASE®	NR	NR	Gastrointestinal Disease, Hypersensitivity Reaction	No	Persistent		NR	NR	Probable/li kely
32	F	CALCITRIOLO EG ®, LAMOTRIGINA DOC®	NR	NR	Hypocalcemia, Convulsive Crisis	Yes	Recovered/resol ved		Positive	NR	Possible
NR	F	NATECAL D3®	NR	NR	Dyspepsia	No	NR		NR	NR	Probable/li kely
55	F	XARENEL®	NR	NR	Nausea	No	Recovered/resol ved		Positive	NR	Probable/li kely
92	F	ROCALTROL®	0	1	Erythroderma	No	NR		Positive	NR	Possible
66	M	DIBASE®	198	198	Nausea, Vomiting	No	Recovered/resol ved		Positive	NR	Possible
78	F	DIBASE®	NR	NR	Pruritus (Therapeutic Error)	No	Persistent		Negative	NR	Possible
NR	F	DIBASE®	195	NR	Dysentery	No	Recovered/resol ved		NR	NR	Possible
46	M	COLECALCIFEROLO, OLUMIANT®, URBASON®, DIBASE®, TONACAL D3®	23	23	Gonarthritis	Yes	Recovering/res olving		Positive	NR	Possible

NR	F	CALCIUM D3 SANDOZ®	90	90	Pruritus, Drug Intolerance	No	NR		NR	NR	Probable/likely
60	F	DIBASE®	0	0	Muscular Pain	No	Recovered/resolved		Positive	Positive	Probable/likely
83	F	CALCIUM SANDOZ®, ROCALTROL®	NR	NR	Confusional State,	Yes	Recovering/resolving		Positive	Negative	Possible
71	M	ROCALTROL ®, RIOPAN®, OSVAREN®	6	0	Dermatitis, Facial Redness, Pruritus	No	Recovered/resolved		Positive	NR	Possible
79	F	DIBASE®	NR	NR	Swelling (Therapeutic Error)	No	NR		NR	NR	Possible
13	M	DIBASE®	0	0	Breathing Difficulty, Pharyngeal Oedema, Rash Trunk	No	Recovering/resolving		Positive	NR	Probable/likely
62	F	COLECALCIFEROLO EG®	NR	0	Confusion	No	Recovered/resolved		Positive	NR	Possible
75	F	DIBASE®	NR	NR	Abdominal Swelling, Constipation (Excl Faecaloma)	No	NR		NR	NR	Probable/likely
63	F	COLECALCIFEROLO, BINOSTO®	NR	20	Constipation	No	Persistent		NR	NR	Possible
71	M	CALCITRIOLO	740	0	Hypercalcaemia, Renal Failure, Infarction	Yes	NR		NR	NR	Possible
NR	F	DIBASE®	NR	NR	Pruritus, Rash	No	Persistent		NR	NR	Probable/likely
70	F	DEDIOL®, CIPRALEX®	NR	73	Abdominal Pain, Epigastric Pain	No	Recovering/resolving		NR	NR	Possible
43	F	XARENEL®	0	0	Urinary Hesitancy, Renal Pain	No	Recovering/resolving		Positive	NR	Probable/likely
60	F	FOSAVANCE®	NR	NR	Decreased Bone Density	No	NR		NR	NR	Possible

63	F	DIBASE®	1	0	Pruritus, Rash, Urticaria	No	Recovering/resolving		NR	NR	Possible
55	F	DIBASE ®, ARAVA®	NR	NR	Retrosternal Pain	No	Recovered/resolved		NR	NR	Possible
57	M	DIBASE®, ZYLORIC®	50	50	DRESS Syndrome	Yes	Recovering/resolving		Positive	NR	Possible
54	F	DIBASE®	5	5	Pruritus, Rash	No	Recovering/resolving		Positive	NR	Probable/likely
52	M	NATECAL D3®	214	30	Muscular Weakness, Pruritus, Rash, Lumbar Pain	No	Persistent		Positive	NR	Probable/likely
14	M	NATECAL D3®	NR	NR	Attention Impaired (Off Label Use)	No	Persistent		NR	NR	Probable/likely
NR	F	FOSAVANCE®, NATEMILLE®	NR	NR	Abdominal Pain, Gastric Pain, Polyuria, Vomiting	No	NR		NR	NR	Possible
72	F	NATECAL D3®	152	NR	Oral Pain	No	Recovered/resolved		Positive	NR	Possible
76	M	NATECAL D3®	NR	NR	Oral Pain (Off Label Use)	No	Recovered/resolved		Positive	NR	Possible
42	F	DIBASE ®, GENVOYA®	NR	NR	Abortion (Pregnancy Exposition)	Yes	Recovered/resolved		NR	NR	Possible
43	F	XARENEL®	0	0	Lumbar Pain, Oligoanuria	No	Recovering/resolving		Positive	NR	Probable/likely
50	M	DIBASE®	NR	NR	Nausea	No	NR		NR	NR	Probable/likely
49	F	DIBASE®	NR	NR	Tachycardia, Throat Tightness	No	Recovered/resolved		Positive	NR	Probable/likely
72	F	CACIT®	NR	0	Epigastric Pain, Hyperidrosis, Hypertension	No	Recovering/resolving		NR	NR	Probable/likely
NR	F	DIBASE®	NR	NR	Nausea	No	NR		NR	NR	Possible
1	M	DIBASE®	NR	0	Somnolence (Unwanted Drug Use)	No	Recovering/resolving		NR	NR	Possible

69	NR	DIBASE®, KOLIBRI®, MEDIPO®, METFONORM®, ZAROXOLYN®, DIURESIX®, MICARDIS®, LETROZOL®, CHLOROQUINE®, OMEPRAZOLO	NR	NR	Urticaria	Yes	NR		NR	NR	Possible
56	F	DIBASE®, NATECAL D3®	797	781	Hydronephrosis, Renal Calculi	Yes	Recovering/resolving		Positive	NR	Possible
56	F	DIBASE. NATECAL D3®	797	781	Hydronephrosis, Renal Calculi	Yes	Recovering/resolving		Positive	NR	Possible
59	F	DIBASE®	9	6	Pruritus	No	Recovered/resolved		Positive	NR	Possible
44	F	DIBASE®	31	31	Cutaneous Rash	No	Recovered/resolved		Positive	NR	Probable/likely
NR	F	COLECALCIFEROLO EG®	NR	NR	Dysentery	No	NR		NR	Positive	Probable/likely
27	F	DIBASE®	NR	4	Acne	No	Recovering/resolving		Positive	NR	Probable/likely
NR	F	DIBASE®, CALCIUM D3 SANDOZ®, LETROZOLO	NR	NR	Diarrhoea	No	NR		NR	NR	Possible
35	M	DIBASE®	NR	1	Muscular Pain, Neuropathy	No	Recovered with sequelae		Positive	Positive	Probable/likely
NR	F	DIBASE®	NR	NR	Mental Confusion, Pruritus	No	NR		NR	NR	Probable/likely
48	F	DIBASE®, SALAZOPYRIN®, EN®, CIPRALEX®	418	418	Cough, Pruritus	No	Persistent		Negative	NR	Possible
68	M	DIBASE®	701	701	Hypercalcaemia	Yes	Recovering/resolving		Positive	NR	Possible
71	F	DIBASE®, ACIDO FOLICO DOC GENERICI®	0	0	Syncope Feeling, Urticaria	Yes	Recovering/resolving		Positive	NR	Possible
1	F	DIBASE®	150	150	Low PTH, Growth Retardation	Yes	Recovering/resolving		Positive	NR	Probable/likely

					(Therapeutic Error)						
81	F	DIBASE®	11	11	Polydipsia (Therapeutic Error)	Yes	Recovering/resolving		Positive	NR	Probable/likely
70	M	DIBASE®	0	0	Eye Burning (Therapeutic Error)	No	Recovering/resolving		Positive	NR	Probable/likely
NR	M	DIBASE®	NR	NR	Hydronephrosis	Yes	Recovering/resolving		Positive	NR	Probable/likely
NR	F	NATECAL D3®	NR	NR	Pruritus	No	Recovered/resolved		Positive	NR	Possible
43	F	DIBASE®	NR	NR	Injaction Site Pain	No	Persistent		Negative	NR	Possible
78	F	NATECAL D3®	NR	NR	Angioedema, Rash	Yes	Recovering/resolving		NR	NR	Probable/likely
NR	F	DIBASE®	NR	NR	Headache, Vomiting	No	Recovering/resolving		Positive	NR	Probable/likely
59	M	ROCALTROL®	144	141	Increased Creatinine, Decreased Appetite	No	Recovered/resolved		Positive	NR	Possible
74	NR	DIBASE®	NR	NR	Increased Prothrombin Time	No	Recovering/resolving		Positive	NR	Possible
47	F	DIBASE®	NR	NR	Epigastralgia, Nausea, Vomiting	Yes	NR		NR	NR	Probable/likely
42	F	COLECALCIFEROLO, NICETILE®	217	217	Renal Colic	No	Recovering/resolving		Positive	NR	Possible
70	M	NATECAL D3®	NR	NR	Feeling Cold, Hyperidrosis	No	Recovered/resolved		NR	NR	Possible
72	F	ZIBENAK®	30	1	Dysentery	No	Recovering/resolving		Positive	NR	Possible
4 Months	F	COLECALCIFEROLO	9	9	Vomiting (Therapeutic Error)	No	Recovering/resolving		Positive	NR	Possible
48	F	DERIL®	NR	NR	Nausea, Vertigo	No	Recovered with sequelae		Positive	NR	Possible

66	F	DIBASE®, ROCALTROL®	265	265	Hypercalcaemia, Hypokinesia, Vertigo, Bradyphrenia (Overdose)	Yes	Recovering/resolving		Positive	NR	Possible
55	M	DIBASE®	264	264	Nausea, Vomiting	No	Recovering/resolving		Positive	NR	Possible
61	M	DIBASE®	0	0	Allergic Urticaria	No	Recovering/resolving		Positive	NR	Probable/likely
70	F	NATECAL D3®	272	271	Diarrhoea, Nausea	No	Recovering/resolving		Positive	NR	Probable/likely
59	F	XARENEL®	5	3	Diarrhoea, Nausea, Vomiting, Dysbiosis	No	Recovered/resolved		Positive	NR	Probable/likely
1 Months	F	DIBASE®	44	44	Hypercalcaemia, Hyperpotassemia (Overdose)	Yes	Recovering/resolving		Positive	NR	Probable/likely
70	M	ROCALTROL®	115	115	Hypercalcaemia	No	NR		NR	NR	Probable/likely
25	F	DIBASE®	NR	NR	Gastric Pain, Asthenia, Dark Urine, Metallic Taste, Nausea, Polydipsia, Polyuria, Anorexia	No	Recovering/resolving		Positive	NR	Probable/likely
59	F	NATECAL D3®	273	242	Dysgeusia	No	Recovering/resolving		Positive	NR	Probable/likely
76	M	XARENEL®, NATECAL D3®	59	15	Sweet Syndrome	No	Recovering/resolving		Positive	NR	Possible
81	M	DIBASE®	NR	881	Weakness, Oral Dryness, Polyuria, Increased Ferritin,	No	Persistent		Negative	NR	Possible
64	F	DIBASE®	NR	NR	Tendon Calcification	Yes	Recovering/resolving		Positive	NR	Probable/likely
71	F	DIBASE®	NR	NR	Erythema	No	NR		NR	NR	Probable/likely

74	M	ROCALTROL®	412	3	Nausea	No	NR		NR	NR	Probable/likely
NR	F	DIBASE®	NR	NR	Skin Reaction	No	NR		NR	NR	Probable/likely
45	F	CACIT®	NR	4748	Dyspepsia	No	Recovered/resolved		NR	NR	Probable/likely
4 Months	F	DIBASE®	55	55	Calcium Increased, Vitamin D Increased (Therapeutic Error)	Yes	Recovering/resolving		Positive	NR	Probable/likely
57	F	DEDIOL	0	1	Nausea, Vertigo (Therapeutic Error)	Yes	Recovering/resolving		Positive	NR	Probable/likely
74	F	NATECAL D3®, BISOPROPOLO TEVA®, XARELTO®	565	427	Gingival Bleeding	No	Recovering/resolving		NR	NR	Possible
44	F	DELTIUS®	23	23	Pruritus, Rash	No	Recovering/resolving		Positive	NR	Probable/likely
80	M	ZIBENAK®	NR	NR	Confusional State, Diarrhoea, Flatulence, Headache, Weakness, Polydipsia, Polyuria, Renal Failure, Somnolence, Thirst, Urticaria, Anorexia	No	NR		NR	NR	Probable/likely
85	F	NATECAL D3®, FOSAVANCE®, ELIQUIS®	1873	1857	Conjunctival Bleeding	No	Recovering/resolving		NR	NR	Possible
79	F	DIBASE®, PRADAXA®, LOSARTAN ACTAVIS®, BISOPROLOLO TEVA®, DOXAZOSINA ALMUS®	473	386	Hematochezia	Yes	NR		NR	NR	Possible
NR	F	CALCIO CARBONATO E VITAMINA D3 DOC GENERICI®	NR	NR	Dyspepsia, Abnormal Drug Taste	No	NR		NR	NR	Probable/likely

76	M	DIBASE®, CLEXANE®, LANSOPRAZOLO ABC®, ALENDRONATO ACTAVIS®, NORMIX®	639	601	Epistaxis	Yes	Recovering/resolving		NR	NR	Possible
92	F	ROCALTROL ®, LASIX®	330	327	Altered State Of Consciousness, Dehydration, Dysarthria, Hypercalcaemia	Yes	NR		NR	NR	Unlikely
33	F	DIBASE®	NR	NR	Abortion Threat	Yes	Recovered/resolved		Positive	NR	Unlikely
74	F	DIBASE®, CLASTEON®	NR	NR	Diarrhoea	No	Persistent		NR	NR	Possible
75	M	DIBASE®, ARIXTRA®, EUTIROX®	624	624	Bloody Stool	No	NR		NR	NR	Possible
38	F	COLECALCIFEROLO EG®	86	0	Arthralgia, Asthenia	No	Persistent		NR	NR	Possible
79	F	DIBASE®, XARELTO®, METHOTREXATE, FOLINA®, CRESTOR®, MEDROL®, PANTOPRAZOLO, SERTRALINA	61	0	Arthralgia	Yes	Recovering/resolving		Positive	NR	Possible
73	M	CALCITRIOLO DOC®, ELIQUIS®, ALLOPURINOLO, LASIX®, NORVASC®, PERINDOPRIL ALMUS®, TAMSULOSINA, CARDIOASPIRIN®, ATENOLOLO, LANSOPRAZOLO	1945	8	Bloody Stool	Yes	Recovering/resolving		NR	NR	Possible
59	F	CALCITRIOLO	NR	NR	Neck Oedema	Yes	Recovered/resolved		Positive	NR	Possible
2 Months	M	DIBASE®	61	61	Hypercalcaemia (Therapeutic Error)	Yes	Recovering/resolving		Positive	NR	Probable/likely
79	F	DIBASE®, CACIT®	51	51	Fatigue, Malaise	No	Recovering/resolving		Positive	NR	Possible

46	M	COLECALCIFEROLO	NR	NR	Arrhythmia	Yes	Recovered/resolved		Positive	NR	Probable/likely
74	F	DIBASE®	NR	NR	Drug Intolerance	No	NR		NR	NR	Probable/likely
6	NR	DIBASE®	NR	NR	Skin Reaction	No	NR		NR	NR	Probable/likely
8	NR	DIBASE®	NR	NR	Skin Reaction	No	NR		NR	NR	Probable/likely
55	F	DIBASE®	NR	NR	Vitiligo Vulgaris	No	NR	BINOSTO®	NR	NR	Possible
4 Months	M	ANNISTER®	31	31	Total Calcium, Ionized Calcium, Sieric Phosphate, Vitamin D Increased (Overdose)	Yes	Recovering/resolving		Positive	NR	Probable/likely
56	F	ANNISTER®	NR	NR	Diffuse Erythema	No	Recovered/resolved		Positive	NR	Probable/likely
69	F	COLECALCIFEROLO	1	0	Abdominal Pain, Sacral Pain, Dysuria, Pruritus	Yes	Recovered/resolved		Positive	NR	Possible
NR	F	DIBASE®	NR	191	Headache, Muscular Weakness	No	Recovering/resolving		Positive	NR	Probable/likely
41	F	CALISVIT®	1	0	Arrhythmia	No	NR		NR	NR	Possible
62	M	COLECALCIFEROLO	NR	0	Abdominal Pain, Urinary Calculus, Hematuria	Yes	Recovering/resolving		Positive	NR	Probable/likely
NR	F	DIBASE®	NR	NR	Choroidal Cystis (Therapeutic Error)	No	Recovered/resolved		Positive	NR	Possible
67	F	DIBASE®, OVIXAN®	NR	NR	Bitter Taste, Palpitations, Head And Throat Tightness, Trichorrhexis,	No	Persistent		NR	NR	Possible

					Eye Burns (Off Label Use)						
70	F	DIBASE®	NR	NR	Asthenia, Mental Confusion, Nausea, Appetite Loss	No	Recovered/resolved		Positive	NR	Possible
62	F	DIBASE®	149	0	Dry Mouth	No	Recovering/resolving		Positive	NR	Probable/likely
78	F	DIBASE®	7	1	Pruritus, Rash	No	Recovered/resolved		Positive	NR	Probable/likely
78	F	DIBASE®, ZOMETA®, REVLIMID®	232	243	Dehiscence	Yes	NR		NR	NR	Possible
41	M	TREDIMIN®	NR	NR	Migraine, Muscular Weakness, Somnolence	Yes	Persistent		Negative	NR	Probable/likely
71	F	COLECALCIFEROLO, CALCITRIOLO	9	9	Asthenia, Hypercalcaemia, Nausea, Pruritus, Postural Instability	Yes	Recovering/resolving		Positive	NR	Possible
72	M	CALCITRIOLO DOC GENERICI®	NR	NR	Cutaneous Rash (Drug Change)	No	Persistent		Negative	Positive	Possible
66	F	NATECAL D3®	44	14	Abdominal Pain, Skin Redness And Pruritus	No	Recovered/resolved		Positive	NR	Probable/likely
52	M	CALCIO CARBONATO E VITAMINA D3 ALMUS®	0	0	Vomiting	No	Recovered/resolved		Positive	NR	Probable/likely
NR	F	XARENEL®	NR	NR	Cutaneous Rash	No	Recovering/resolving		NR	NR	NR
83	M	COLECALCIFEROLO, ELIQUIS®, RAMIPRIL ACTAVIS®, LANSOPRAZOLO ABC®, GABAPENTIN ABC®, INSULINA	NR	NR	Stroke	Yes	NR		NR	NR	Possible

21 Days	F	DIBASE®	NR	NR	Insomnia, Psychomotor Hyperactivity	No	Recovering/resolving		NR	NR	Possible
27	F	DIBASE®, ZITROMAX®	5	1	Stomach Cramps, Diarrhea	No	Recovered/resolved		Positive	NR	Possible
61	M	DIBASE®, UNIXIME®, DELTACORTENE®	5	3	Stomach Cramps, Diarrhea	No	Recovered/resolved		Positive	NR	Possible
84	M	DIBASE®, ROCEFIN®, URBASON®, CLEXANE®	9	3	Mental Confusion, Disorientation, Dizziness	No	Recovered/resolved		Positive	NR	Possible
60	F	LAMPARD®	NR	NR	Dizziness (Drug Error Use)	No	NR		NR	NR	Probable/likely
NR	F	NATECAL D3®	NR	NR	Scalloped Tongue, Altered Taste	No	Recovered/resolved		Positive	NR	Probable/likely
77	F	DIBASE®, COUMADIN®, EUTIROX®, FUROSEMIDE FISIOPHARMA®, AMIODARONE CLORIDRATO BIOINDUSTRIA LIM®, BISOPROLOLO TEVA®, CARDIOASPIRIN®, ALLOPURINOLO TEVA ITALIA®, ATORVASTATINA AUROBINDO ITALIA®	155	136	Hemorrhoidal Bleeding	No	Recovered/resolved		NR	NR	Possible
4 Months	M	COLECALCIFEROLO	131	131	Restlessness, Vomiting	No	Recovering/resolving		Positive	NR	Probable/likely
32	M	DIBASE®	0	0	Angioedema	No	Recovered/resolved		Positive	NR	Probable/likely
85	F	ADISTEROLO	NR	NR	Hypervitaminosis D (Drug Abuse, Off Label Use)	No	NR		NR	NR	Possible

NR	F	CALCIO CARBONATO/COLECALCIFEROLO	34	NR	Oropharingeal Pain	No	Recovering/resolving		Positive	NR	Probable/likely
56	F	DIBASE®	NR	NR	Weakness, Fatigue, Headache, Hyperidrosis, Fever	No	NR		NR	NR	Possible
19	F	DIBASE®, SERTRALINA®, OLANZAPINA	NR	NR	Abdominal Pain, Cold Sensation, Headache, Pyrexia	No	Persistent		Negative	NR	Possible
NR	M	COLECALCIFEROLO MYLAN®	NR	NR	Gastric Pain (Intentional Wrong Use, Expired Drug, Missed Dose)	No	NR		NR	NR	Probable/likely
59	F	NODIGAP®	NR	NR	Dry Eye, Headache, Rash	No	Persistent		Negative	NR	Probable/likely
40	F	COLECALCIFEROLO	0	3	Cutaneous Rash	No	Persistent		Negative	NR	Probable/likely
66	F	COLECALCIFEROLO	0	0	Tachycardia, Vomiting, Diarrhoea	Yes	Recovered/resolved		Positive	NR	Probable/likely
72	M	CALCITRIOLO TEVA®, TARDYFER®	NR	NR	Hard Stool, Decreased Urine Flow	No	NR		NR	NR	Possible
80	F	DIBASE®, COMIRNATY®	0	0	Injection Site Oedema	No	Recovered/resolved		Positive	NR	Possible
75	M	DIBASE®	0	0	Angioedema	No	Recovered/resolved		Positive	NR	Possible
33	M	DIBASE®	6	6	Nausea (Administration Error)	No	Persistent		Negative	NR	Probable/likely
70	F	DIBASE®	558	47	Granuloma Anullare	No	Persistent		Negative	NR	Probable/likely
1 Months	M	DIBASE®	71	0	Nephrocalcinosis (Administration Error)	Yes	Persistent		Negative	NR	Probable/likely

70	M	CALCITRIOLO DOC GENERICI®, ALLOPURINOLO ACCORD®, ALFUZOSINA AUROBINDO®	394	394	Pruritus	No	Persistent		NR	NR	Possible
47	F	DIBASE®, BARICITINIB	NR	NR	Acne, Cough, Arm Pain, Rhinorrhoea	No	Persistent		Negative	NR	Possible
62	M	COLECALCIFEROLO	0	0	Skeletal Pain, Impaired Breathing, Allergic Reaction, Lymphadenopathy, Muscular Pain, Tachycardia	Yes	Recovering/resolving		Positive	NR	Probable/likely
61	F	XARENEL®, VAXZEVRIA®	0	16	Fatigue, Pruritus Rash, Somnolence	No	Recovered/resolved		NR	NR	Possible
65	F	NATECAL D3®	NR	NR	Wheals	No	Recovered/resolved		NR	NR	Possible
66	F	OROTRE®	NR	NR	Ischaemic Colitis, Costipation (Excl Faecaloma)	Yes	Recovered/resolved		NR	NR	Probable/likely
63	F	DIBASE®	NR	NR	Congiuntivitis, Fever, Gastroenteritis, Leukopenia, Lymphopenia, Oropharyngeal Pain, Nausea, Thrombocytopenia	No	Recovered/resolved		NR	NR	Possible
23	F	DIBASE®	0	0	Fatigue	No	Recovering/resolving		NR	NR	Probable/likely
29	F	DIBASE®	NR	NR	Renal Pain, Somnolence	No	NR		NR	NR	Possible

82	F	ADROVANCE®	NR	NR	Jaw Osteonecrosis	Yes	NR		NR	NR	Probable/likely
59	F	COLECALCIFEROLO	NR	NR	Pyrexia, Fatigue	No	Recovered/resolved		NR	NR	Possible
60	F	ACIDO ALENDRONICO SALE SODICO TRIIDRATO, COLECALCIFEROLO	7	6	Vertebral Pain, Knee Pain	No	Persistent		NR	NR	Probable/likely
73	F	FOSAVANCE®	0	0	Vision Loss	No	Recovered/resolved		NR	NR	Possible
43	F	ADROVANCE®	0	0	Pain, Migraine, Fever, Nausea, Vomiting, Drug Intolerance	No	Recovered/resolved		NR	NR	Possible
84	M	CERNEVIT®	1	1	Rash	No	Recovered/resolved		Positive	NR	Possible
35	F	COLECALCIFEROLO	105	103	Cutaneous Rash, Fever	No	Recovered/resolved		Positive	Positive	Probable/likely
74	F	DIIDROGYL®	NR	NR	Bitter Taste	No	Persistent		NR	NR	Possible
80	F	NATECAL D3®	NR	NR	Skin Pain, Hemorrhoids, Fissures, Constipation	No	NR		NR	NR	Probable/likely
29	F	DIBASE®	0	0	Abdominal Pain, General Malaise	No	Recovered/resolved		NR	NR	Probable/likely
53	F	COLECALCIFEROLO	NR	NR	Drug Intolerance, Formication, Syncope Feeling, Tachycardia	Yes	Recovering/resolving		NR	NR	Possible
29	F	NODIGAP®	NR	NR	Dermatitis, Wheals, Pruritus	No	Persistent		NR	NR	Possible
67	F	FOSAVANCE®	5964	5978	Jaw Osteonecrosis	Yes	Recovered/resolved		NR	NR	Probable/likely
20	F	XARENEL®	NR	NR	Urticaria	No	Recovered/resolved		NR	NR	Probable/likely
NR	F	NATECAL D3®	NR	NR	Migraine With Aura	No	Recovered/resolved		Positive	Positive	Probable/likely

72	M	CERNEVIT®	NR	NR	Advanced Cancer, Hypertensive Crisis, Disease, Stiffness	Yes	Fatal		NR	NR	Possible
85	F	COLECALCIFEROLO	NR	NR	Abdominal Pain, Vomiting	No	Persistent		NR	NR	Possible
59	F	COLECALCIFEROLO	1	0	Confusion, Abdominal Pain, Nausea	No	Recovering/resolving		Positive	NR	Probable/likely
50	M	COLECALCIFEROLO	NR	NR	Calcium Intoxication, Increased Vitamin D (Overdose)	Yes	Recovering/resolving		NR	NR	Probable/likely
56	F	DIBASE®	0	1	Intestinal Dysbiosis, Functional Gastrointestinal Disease, Colon Diverticulosis, Visceral Oedema, Intestinal Obstruction	Yes	Recovered with sequelae		Positive	NR	Possible
70	F	DERIL®	NR	NR	Hypercalcaemia	Yes	Recovered/resolved		Positive	NR	Possible
62	F	NATECAL D3®	NR	NR	Flatulence	No	Recovered/resolved		NR	NR	Possible
31	F	COLECALCIFEROLO	21	12	Pruritus	No	Recovering/resolving		Positive	NR	Possible
57	F	DIBASE®	0	0	Skin Formication, Angoedema, Pruritus	Yes	NR		Positive	NR	Probable/likely
80	M	COLECALCIFEROLO	NR	NR	Lower Limb Myalgia (Drug Abuse)	Yes	Persistent		Positive	NR	Possible

11 Months	F	COLECALCIFEROLO	336	337	Hypercalcaemia, Hyperphosphataemia	No	Recovering/resolving		Positive	NR	Probable/likely
85	M	OROTRE®, ROCALTROL®	NR	NR	Hypercalcaemia	Yes	Recovered/resolved		NR	Negative	Possible
72	F	DIBASE®	NR	NR	Facial Cutaneous Rash	No	Recovered/resolved		NR	NR	Probable/likely
80	M	ANNISTER®	15	15	Cutaneous Rash, Pruritus	Yes	Recovered/resolved		Positive	NR	Possible
37	M	FOSAVANCE®	5	5	Lombar Pain, Acute Renal Failure	Yes	Persistent		NR	NR	Possible
75	F	NATECAL D3®	NR	NR	Walking Difficulty, Musculoskeletal Stiffness	No	Recovering/resolving		NR	NR	Probable/likely
31	F	DIBASE®	81	81	Increased Potassium	No	Recovering/resolving		Positive	NR	Probable/likely
83	F	FOSAVANCE®	30	10	Dizziness, Renal Pain, Leg Pain, Fatigue, Paresthesia	No	Recovered with sequelae		NR	NR	Probable/likely
2 Months	M	DIBASE®	NR	NR	Hyperphosphataemia, Hypervitaminosis D (Therapeutic Error)	No	Recovered/resolved		Positive	NR	Probable/likely
44	F	COLECALCIFEROLO	0	2	Urticaria	Yes	Recovering/resolving		NR	NR	Possible
44	F	DIBASE® , OROTRE® , NEODIDRO®	NR	NR	Constipation	Yes	Persistent		NR	NR	Possible
52	F	NEODIDRO®	4	6	Dark Urine, Dizziness, Epileptic Crisis, Syncope, Tremor, Asthenia (Overdose)	Yes	NR		NR	NR	Possible
70	M	XARENEL®	69	0	Vertigo	No	Recovered/resolved		Positive	NR	Probable/likely

35	F	NODIGAP®	0	0	Rash, Rash, Pruritus, Redness	No	Recovered/resolved		NR	NR	Probable/likely
54	F	DIBASE®	0	0	Erythema	Yes	Recovering/resolving		Positive	NR	Probable/likely
53	M	COLECALCIFEROLO	NR	NR	Worsening, Nighmares, Sleep Disturbance	No	NR		NR	NR	Possible
73	F	NODIGAP®	0	16	Urticaria, Pruritus, Constipation (Excl Faecaloma)	No	Persistent		NR	NR	Probable/likely
82	F	ADROVANCE®	0	7213	Jaw Osteonecrosis	Yes	NR		NR	NR	Possible
79	F	VANTAVO®	0	0	Dysgeusia, Abdominal Pain, Fever, Abdominal Swelling, Nausea, Vomiting	No	Recovered/resolved		NR	NR	Possible
60	F	ADROVANCE®	0	7	Abdominal Cramps, Gastritis	No	Recovered/resolved		NR	NR	Possible
53	F	DIBASE®	NR	NR	Dizziness	No	Recovered/resolved		NR	NR	Possible
58	F	ANNOVA®	0	14	Angioedema	No	Recovering/resolving		Positive	NR	Possible

NR: not reported

Supplementary Table 3. ARs by PT experienced by patients using concomitant medicines

**Concomitant drugs**	**Adverse Reactions**	
	**PT**	**Serious / not serious** (n)
**Thiazide diuretics**	Dermatitis/erythema	0 / 2
	Abdominal/gastric pain, diarrhoea	0 / 5
	Others	0 / 7
**Digoxin**	Hypercalcemia	1 / 1
	Dermatitis/erythema	0 / 2
	Others (excluded cardiac disorders)	1 / 2
**Warfarin**	Increased INR	1 / 12
	Increased prothrombin time	1 / 0
	Decreased INR	1 / 2
	Alterated blood pressure	0 / 1
	Hemorrhoidal bleeding	0 / 1
	Hematoma	0 / 1
	Others	1 / 4

Supplementary Table 4. Comparison ARs distributions by System Organ Class (SOC) between pre-COVID-19 (period 1: 2017–2019) and during the COVID-19 pandemic (period 2: 2020–2022).

**Disorders (SOC)**	**Period 1**		**Period 2**		***p*-Value**
	N	%	N	%	*p*
Gastrointestinal	77	31.3	96	36.2	0.2
Skin and subcutaneous tissue	52	21.1	45	17.0	0.2
General	14	5.7	30	11.3	**0.02**
Investigations	15	6.1	18	6.8	0.8
Renal and urinary	10	4.1	7	2.6	0.4
Nervous system	21	8.5	31	11.7	0.2
Vascular	8	3.3	3	1.1	0.09
Respiratory	8	3.3	4	1.5	0.2
Cardiac	5	2.0	4	1.5	0.4
Musculoskeletal and connective	10	4.1	14	5.3	0.5
Metabolism and nutrition	9	3.7	0	0.0	**0.001**
Psychiatric	12	4.9	5	1.9	0.05
Ear and labyrinth	2	0.8	2	0.8	0.6
Eye	2	0.8	4	1.5	0.3
Immune system	1	0.4	3	1.1	0.4
Hepatobiliary	0		1	0.4	0.5
Total	246		265		0.01

Supplementary Table 5. ARs distributions by System Organ Class (SOC) for each year in pre-COVID-19 (2017–2018-2019).

Disorders (SOC)	ARs n (%)					
	2017		2018		2019	
	Serious	Not serious	Serious	Not serious	Serious	Not serious
Gastrointestinal	4 (40.0)	13 (38.2)	1 (4.2)	27 (37.5)	3 (8.8)	29 (41.4)
Skin and subcutaneous	-	7 (20.6)	5 (20.8)	24 (33.3)	6 (17.6)	10 (14.3)
General and administration site	2 (20.0)	3 (8.8)	1 (4.2)	3 (4.2)	-	5 (7.1)
Investigations	-	-	3 (12.5)	1 (1.4)	7 (20.6)	4 (5.7)
Renal and Urinary	-	-	2 (8.3)	2 (2.8)	2 (5.9)	4 (5.7)
Nervous system	-	4 (11.8)	4 (16.7)	7 (9.7)	5 (14.7)	1 (1.4)
Vascular	-	-	-	1 (1.4)	6 (17.6)	1 (1.4)
Respiratory	-	2 (5.9)	2 (8.3)	2 (2.8)	-	2 (2.9)
Cardiac	-	1 (2.9)	1 (4.2)	-	1 (2.9)	2 (2.9)
Musculoskeletal	2 (20.0)	1 (2.9)	2 (8.3)	2 (2.8)	-	3 (4.3)
Metabolism and nutrition	-	1 (2.9)	1 (4.2)	-	3 (8.8)	4 (5.7)
Psychiatric	2 (20.0)	2 (5.9)	2 (8.3)	3 (4.2)	-	3 (4.3)
Ear and labyrinth	-	-	-	-	1 (2.9)	1 (1.4)
Immune system	-	-	-	-	-	1 (1.4)
Hepatobiliary	-	-	-	-	-	-
Total	10	34	24	72	34	70

Supplementary Table 6. ARs distributions by System Organ Class (SOC) for each year during the COVID-19 pandemic (2020–2021-2022).

Disorders (SOC)	ARs n (%)					
	2020		2021		2022	
	Serious	Not serious	Serious	Not serious	Serious	Not serious
Gastrointestinal	4 (14.8)	23 (40.4)	10 (33.3)	33 (44.6)	1 (5.9)	25 (43.1)
Skin and subcutaneous	2 (7.4)	14 (24.6)	5 (16.7)	13 (17.6)	3 (17.6)	8 (13.8)
General and administration site	2 (7.4)	4 (7.0)	-	14 (18.9)	4 (23.5)	6 (10.3)
Investigations	7 (25.9)	3 (5.4)	1 (3.3)	1 (1.4)	1 (5.9)	5 (8.6)
Renal and Urinary	3 (11.1)	-	1 (3.3)	-	2 (11.8)	1 (1.7)
Nervous system	3 (11.1)	7 (12.3)	2 (6.7)	10 (13.5)	3 (17.6)	6 (10.3)
Vascular	1 (3.7)	1 (1.8)	1 (3.3)	-	-	-
Respiratory	2 (7.4)	1 (1.8)	1 (3.3)	-	-	-
Cardiac	-	1 (1.8)	3 (10.0)	-	-	-
Musculoskeletal	2 (7.4)	1 (1.8)	4 (13.3)	2 (2.7)	2 (11.8)	3 (5.2)
Metabolism and nutrition	-	-	-	-	-	-
Psychiatric	1 (3.7)	1 (1.8)	-	1 (1.4)	-	2 (3.4)
Ear and labyrinth	-	1 (1.8)	-	-	-	1 (1.7)
Immune system	-	-	1 (3.3)	-	1 (5.9)	1 (1.7)
Hepatobiliary	-	-	1 (3.3)	-	-	-
Total	27	57	30	74	17	58
